# Minimal organism JCVI-syn3.0, an engineered derivative of *Mycoplasma mycoides*, encodes an assortment of unrecognized membrane transporters and immunity factors

**DOI:** 10.1128/msphere.00077-26

**Published:** 2026-08-28

**Authors:** Arcady R. Mushegian, Phillip K. Yamamoto, Naoki Torikai, Kazuharu Arakawa

**Affiliations:** 1 Division of Molecular and Cellular Biosciences, National Science Foundation, Alexandria, Virginia, USA; 2 Institute for Advanced Biosciences, Keio University 74060, Tsuruoka, Yamagata, Japan; 3 Systems Biology Program, Graduate School of Media and Governance, Keio University 318527, Fujisawa, Kanagawa, Japan; 4 Integrative Genetics and Genomics Program, University of California Davis 8789, Davis, California, USA; 5 Faculty of Environment and Information studies, Keio University 120723, Fujisawa, Kanagawa, Japan; Third Institute of Oceanography Ministry of Natural Resources, Xiamen, China

**Keywords:** minimal genome, Cas4, JCVI-Syn3.0, bacterial immunity, *Mycoplasma mycoides*

## Abstract

In 2016, researchers at the J. Craig Venter Institute produced JCVI-Syn3.0, a derivative of *Mycoplasma mycoides* subsp. *capri* Large Colony strain GM12 with a re-engineered, minimized genome. The minimal synthetic genome of 532 kbp encodes 473 genes, selected from ∼910 parent genes on the basis of their indispensability, that is, a lethal phenotype caused by a knockout of each such gene. Among these genes, 438 genes encode proteins, of which ∼150 were not assigned a molecular function or predicted cellular role. A decade later, despite community annotation efforts, 50–60 of these putative proteins still lack any assigned function. We analyzed the intrinsic sequence characteristics, evolutionary conservation, and three-dimensional AlphaFold models of this least understood subset of the JCVI-Syn3.0 genes. We predicted conserved sequence motifs and structural features and inferred molecular functions of six proteins likely involved in membrane transport or membrane interactions, and six proteins that apparently belong to bacterial immunity systems, such as putative antitoxins, an essential toxin-like protein, and a member of a previously unnoticed clade of Cas4-like nucleases. The spatial models produced for this challenging data set by the AlphaFold pipeline help dissect proteins into discrete domains, which are often more convenient to analyze by sequence similarity than whole proteins. In several cases, AlphaFold models also enable functional inference more directly, based on spatial matching of the models to the databases of known protein structures.

**IMPORTANCE:**

The smallest synthetic genome, JCVI-Syn3.0, encodes 438 proteins. JCVI-Syn3.0 was obtained by combining all genes whose deletion from a larger parental genome of *M. mycoides* subsp. *capri* Large Colony strain GM12 causes a complete stop or a significant delay in bacterial growth. Thus, nearly every protein in this minimal genome is essential; however, we do not know what ∼15% of these genes do. Using computational methods, we predicted molecular functions for about a quarter of these enigmatic proteins, including several proteins that are related to the components of recently discovered bacterial immunity systems.

## INTRODUCTION

A minimal cell is an organism that contains only the genetic loci strictly necessary for its autonomous growth in a defined environment, such as a nutrient-rich laboratory medium (1). The set of all such genes in a given species is called “a minimal genome” for that species. The content of a minimal genome depends on the composition of the growth medium and other environmental parameters: the number and identity of genes in a minimal genome may change if the environment changes (2). Minimal genomes are seen as a vehicle for the discovery and testing of basic engineering principles that facilitate cellular life in the hope of “achiev[ing] a complete understanding of what it takes to be alive” (3).

Minimal genomes also may be more tractable by mathematical modeling than larger genomes because many properties of metabolism, such as the complexity of molecular interaction landscapes or the number of possible “on-off” gene switch configurations, must scale non-linearly with the number of genes and gene products.

In practice, minimal genomes are usually designed by removing genes from species whose genomes are already small, commonly from bacteria. In the middle of the last century, Harold Morowitz argued that mycoplasmas, with their tiny cell size, lack of cell wall, and low DNA-per-cell content, may be the simplest form of cellular life capable of growing autonomously in defined rich media (4–6). One of the species, *Mycoplasma genitalium*, was the second organism whose genome was completely sequenced (7), and its genome, consisting of 580,076 DNA base pairs and encoding 476 proteins, remains the smallest one known for naturally extracellularly living, autonomously replicating bacteria. Near-saturation transposon mutagenesis in *M. genitalium* and a comparison to a similarly mutagenized larger genome of related *M. pneumoniae* had suggested 265–351 essential genes (8). Modified mutagenesis protocols and more comprehensive screening provided a revised estimate of 370 essential genes in *M. genitalium* (9).

The subsequent experiments used a mycoplasma species more amenable to the whole-genome manipulations, *M. mycoides* subspecies *capri* Large Colony (LC) strain GM12 (see Materials and Methods for more on the taxonomy of this organism). First, a completely synthetic genome of *M. mycoides* with almost wild-type gene content was created at the J. Craig Venter Institute (10). The genome, JCVI-Syn1.0 with 901 genes, was mutagenized with transposon cassettes, and inactivating insertions were mapped to 432 genes. This information was used to design and test two reduced genomes, JCVI-Syn2.0 with 516 genes and JCVI-Syn3.0 with 438 protein-coding and 35 RNA-coding genes (11). The latter genome was assembled from oligonucleotides into a circular chromosome and introduced into the cytoplasm of a related mycoplasma species, where, aided by a genetically encoded selection marker, it directed cell reproduction with a long doubling time of ∼180 min, compared to ∼60 min in the wild type. JCVI-Syn3.0 remains the smallest bacterium capable of surviving in a rich cell-free medium. More details on the design, assembly, and testing of JCVI-Syn3.0 can be found in a previous study (11); also see previous studies (12–15) for additional discussions of further modified strains JCVI-Syn3A (a strain with a few gene additions and a doubling time of ~120 min) and JCVI-Syn3B. The latter two genomes are not considered in detail here, except to say that Syn3A has been re-mutagenized by transposons, and on the basis of the insertion tolerance, all genes were re-classified as essential (no insertions in a gene are tolerated), quasi-essential (transposon insertion is not lethal but causes growth disadvantage), and, in a few cases, non-essential (13). We will discuss the implications of this when talking about the individual genes.

More than 240 protein-coding genes in JCVI-Syn3.0 have high sequence similarity to well-characterized gene products in sequence databases, allowing genome annotation, prediction of gene functions, whole cell metabolic reconstruction, and flux balance analysis modeling (12). However, for 84 gene products in JCVI-Syn3.0, functional prediction could be made only at a generic level, for example, suggesting the class of enzymatic reaction catalyzed by a protein, but not its exact molecular function or biological role. Furthermore, the functions of ∼65 gene products were completely unknown; in the best case, some of them could be assigned to a conserved protein family that had no functional annotation in the databases.

In the following years, growing knowledge of the functional needs of gram-positive bacteria, to which mycoplasmas are phylogenetically related, allowed researchers to make functional assignments for several dozen of those essential but enigmatic proteins. Some of them provided the putative “missing links” in the reconstructed metabolic map of JCVI-Syn3.0/Syn3A, while others are likely to perform certain initially overlooked functions, such as the deactivation of toxic small molecules resulting from metabolic errors or the removal of damaged proteins and RNAs (16–18). However, the functions of 50–60 protein-coding genes in JCVI-Syn3.0 were still not predicted.

In this work, we inferred the molecular functions of this least understood part of the JCVI-Syn3.0 proteome. We predicted various properties of the putative proteins, matched their sequences to the ever-growing collections of conserved sequence domains, and compared their three-dimensional structures, recently predicted by the AlphaFold algorithm, to the database of known protein spatial structures. Our analysis provided novel functional annotations for a sizable portion of the “dark matter” encoded by this genetically minimal organism.

## MATERIALS AND METHODS

### Data sets

The genome of *M. mycoides* subspecies *capri* LC strain GM12 (GenBank accession CP001668.1) encodes 829 protein-coding genes (19). The organism was originally classified as *M. mycoides* subspecies *mycoides*, Large Colony strain GM12 in 1983 (20) but was reclassified as a serovar of subspecies *capri* in 2009 (21). The latter classification is accepted in the National Center of Biotechnology Information (NCBI) Taxonomy database and is useful for data retrieval. In the following, *M. mycoides* refers to this genetic background. We do not take a position on the proper taxonomy of the organism. This protein set is called Set A in this paper. The collection of all 438 protein-coding genes in JCVI Syn3.0 (11) is called Set B; it is accessible as the set of proteins linked to GenBank entry CP014940.1. The collection of 56 genes in JCVI-Syn3.0 that remained without functional annotation after the 2016 analysis was obtained as follows. The database of genes and their functions, available as a supplemental file in the study by Hutchison et al. (11), was filtered by the value in column AI, retaining only genes whose annotation was completely uninformative (typically, “hypothetical protein,” “putative protein,” or a member of a functionally uncharacterized family in the European Bioinformatics Institute protein family database PFAM). The gene products whose function was not predicted in the study by Hutchison et al. but was inferred in the study by Danchin and Fang (16) (their Table S1) were removed from the list next. The remaining set of 56 genes is called Set C. Obviously, Set C is a subset of Set B, and Set B is a subset of Set A. GenPept identifiers for the proteins in Set C are listed in Table S1.

Transmembrane regions in protein sequences were predicted using the web version of the DeepTMHMM 1.0 algorithm with default parameters (22). Signal peptides were predicted using the SignalP 6.0 web server (23); proteins marked “SP,” “LIPO,” “TAT,” “TATLIPO,” or “PILIN” were considered secreted. The regions of intrinsic disorder in protein sequences were predicted with the IUPred3 web server (24). Protein coverage by conserved sequence domains was assessed by parsing the GenPept entries for each protein; this considers only the domains already annotated in the NCBI Conserved Domains Database. Due to significant deviations from normality in all distributions (assessed using the Shapiro-Wilk test), statistical differences between Sets A, B, and C were evaluated using the Kruskal-Wallis test, followed by Dunn-Bonferroni correction. Counts and length distributions of all the features listed above were summarized using *ad hoc* Python code.

Sequence similarities between the Set C proteins from JCVI-Syn3.0 and the database domain families were evaluated using the HHPred server (25), with the default search mode (local similarity and no realignment). The searches were initially run in October 2023; as the *z* scores and *E*-values of the HHPred search depend on the size and complexity of the search space, the searches were repeated periodically, and updated values were collected. The values in the text refer to searches against PDB_mmCIF70 of March 30, 2025 and NCBI_ConservedDomains(CD) v3.19. Sequence alignments were either imported directly from the HHPred outputs or performed with the MUSCLE5 program (26).

The models for all *M. mycoides* proteins, predicted with the AlphaFold2-Monomer pipeline in 2022 (“AlphaFold” in the rest of the paper), were obtained from the AlphaFold Protein Structure Database at the European Bioinformatics Institute (27). We also predicted some structures using ColabFold v1.5.2 (28). The spatial structures of the AlphaFold models were matched to the structures in the Protein Data Bank (PDB) (29) using the Dali server (30). Protein structures were visualized and analyzed using the open-source version of PyMOL software (31).

## RESULTS

### The “Set C” proteins are shorter on average than the *M. mycoides* and JCVI-Syn3.0 proteins, are less conserved in evolution, and are enriched in helices and membrane segments

**Fig 1:**
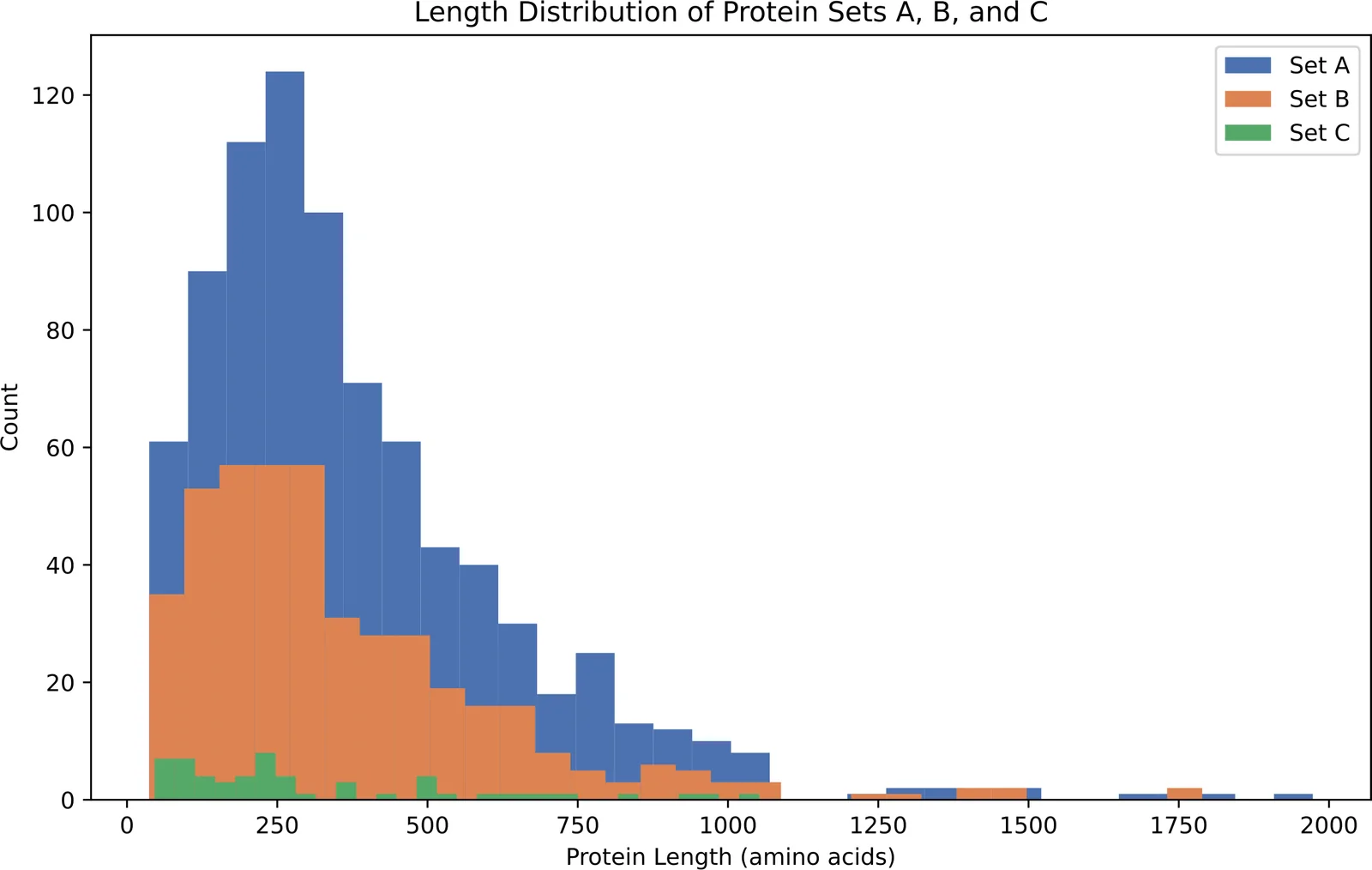
Distribution of sequence lengths in proteins encoded by the JCVI-Syn3.0 genome and in its subsets.

We calculated several descriptive statistics for the protein sequences in JCVI-Syn3.0 to see whether proteins in Set C differ significantly from the genome as a whole. First, we compared the lengths of the protein sequences in Sets A, B, and C (Fig. 1). The proteins in Set C tend to be shorter than the proteins in the other two sets; in particular, Set C is depleted of extremely long proteins, with only three proteins longer than 750 amino acids, compared to dozens of such proteins in Sets A and B.

**Fig 2:**
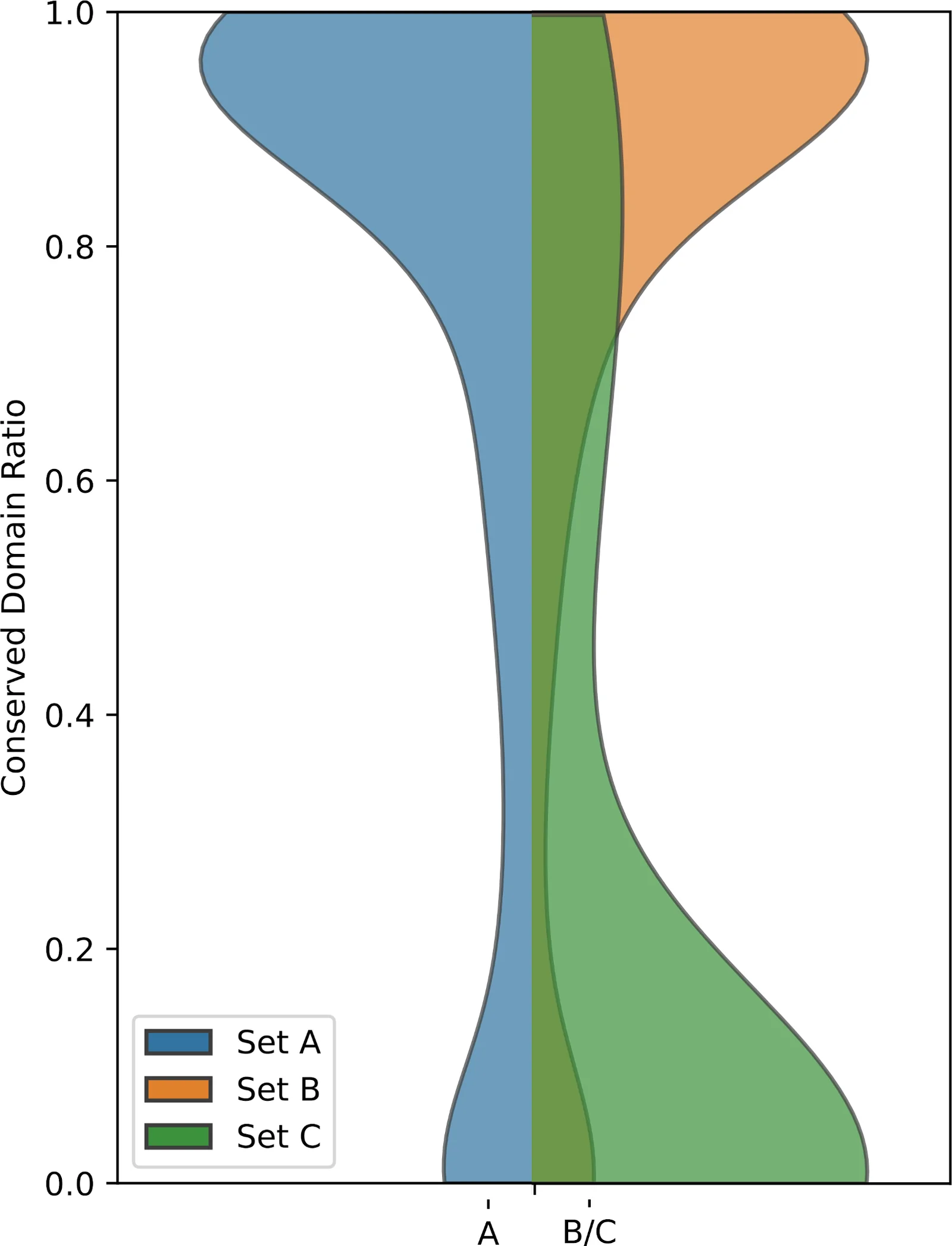
Coverage of proteins in Sets A, B, and C by conserved domains from the NCBI CDD database. Conserved domain ratio (the *Y*-axis) is the ratio of the number of amino acids within annotated conserved domains and the number of amino acids in the entire protein.

We then studied the representation of the known evolutionarily conserved protein sequence domains in the three sets. We found that in Set A, 13.6% of proteins contained no recognizable conserved domains, 74.3% contained one domain, and 12.1% contained two or more conserved domains. In Set B, the corresponding numbers were similar (10.7%, 81.7%, and 7.6%, respectively). In Set C, however, they were very different; –60.7% had no recognizable domains, only 37.5% contained one conserved domain, and just ∼2% (i.e., one protein) contained two or more domains. These lower proportions of conserved domains are also reflected in the downward shift in the fraction of protein length covered by conserved domains in Set C (Fig. 2). Statistical comparison of the conserved domain content in three sets showed significant differences across all three sets (Kruskal-Wallis *P* < 10^−16^). Pairwise comparisons confirmed that Sets A, B, and C all differ significantly from one another (Dunn-Bonferroni adjusted *P* < 0.01 for all pairs).

We wondered whether the low coverage of Set C proteins by recognized conserved domains could be explained in part by the difference in their structural properties from Sets A and B. To investigate this, we first predicted the regions of structural disorder within the three sets; disordered regions are less well conserved in evolution than well-structured ones (32), and their prevalence would correlate with low coverage by conserved domains. The results are shown in Table 1. They suggest that Set C proteins are neither dramatically depleted nor enriched in structurally disordered regions compared to the other two sets.

**TABLE 1:** Percentages of proteins with a given fraction of residues within disordered regions

Set	0–5	5.1–20	20.1–50	50.1–100
A	80	11.1	7.3	1.5
B	85.2	9.3	4.3	1.1
C	81	13.8	3.5	1.7

Next, we took advantage of the structural models for all *M. mycoides* proteins built with the AlphaFold algorithm and asked how different types of secondary structure are represented in three sets. The results, shown in Table 2, indicate that nearly the same percentages of residues in all three sets fall into loops and unstructured regions, but the distribution of residues between predicted alpha-helices and beta-strands in Set C is quite different from the other two sets. In Sets A and B, the percentage of residues in strands is nearly the same (16%–17%), but in Set C, it is only about half of that, whereas the proportion of residues in helices is accordingly higher in Set C than in the other two sets.

**TABLE 2:** Percentages of residues within different types of secondary structures

Set	Helices	Strands	Loops
A	46.4	15.9	37.3
B	45.3	17.3	37.4
C	57.1	8.5	34

**Fig 3:**
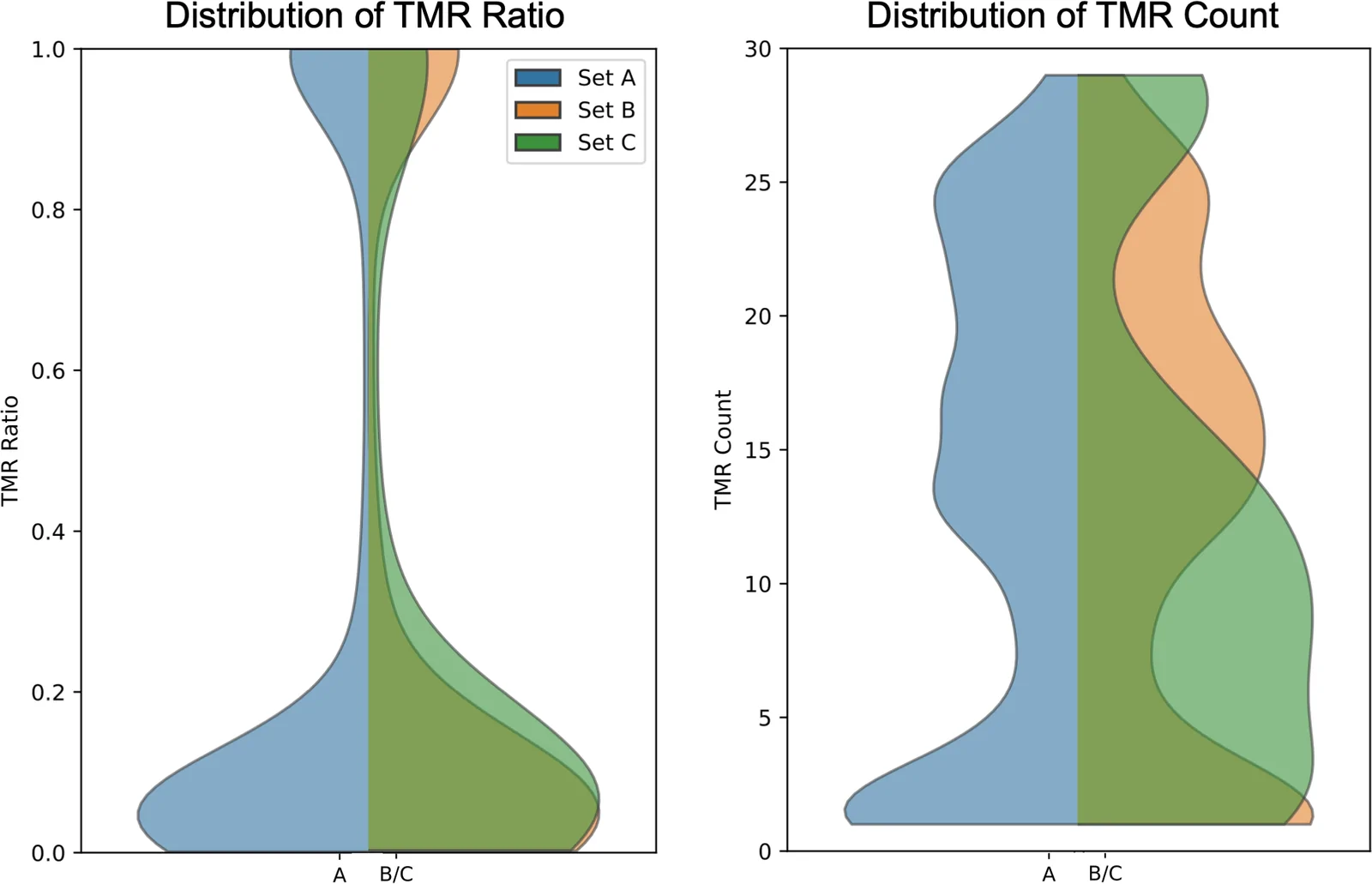
Transmembrane regions predicted by DeepTMHMM. (Left) The fraction of residues in transmembrane helices (TMR ratio). (Right) The number of TMRs per protein.

We then asked whether the higher helix content in Set C proteins could be explained by the prevalence of helical transmembrane regions. Using the Hidden Markov Models-based approach, we evaluated the distributions of proteins in the three sets by the proportion of their lengths covered by transmembrane segments and by the number of such segments per molecule. The results indicate that the predicted transmembrane segments do not seem to account for the larger fraction of residues within helical regions in Set C (Fig. 3). However, significant differences were observed between total helix counts in Sets A, B, and C (Kruskal-Wallis *P* = 0.0034), and within the Set A-Set C pair (*P* = 0.012). Transmembrane segment count in Set C was also appreciably lower than that in Set A (*P* < 0.001).

We also predicted the putative N-terminal leader peptides that mark secreted or extracellular, membrane-retained proteins. The fractions of proteins with predicted signal peptides in Sets A, B, and C were 11.9%, 3.4%, and 7.1%, respectively, indicating that secreted proteins are depleted from the minimal genome (perhaps because many such proteins are involved in microbe-host interactions irrelevant to growth on the rich medium) but overrepresented in its least-annotated portion.

### Membrane-associated proteins with recognizable functions: transporters and small-molecule interactors

Using the HHPred algorithm, we scanned the conserved domain databases with Hidden Markov Models of each Set C protein. We also examined the three-dimensional structures predicted by AlphaFold and used the Dali algorithm to compare them with the database of experimentally determined protein structures. To predict transmembrane regions and the loops between them, we used the DeepTMHMM program. There were some differences in the AlphaFold and DeepTMHMM predictions for the same protein. In particular, we observed that in the AlphaFold models, closely spaced helices were sometimes merged into one long helix, perhaps because the algorithm did not consider protein-membrane interactions. In contrast, DeepTMHMM takes into account the knowledge of sequence signatures to infer direction-reversing turns and other features of the membrane topology (22).

In total, Set C was predicted to contain 20 proteins with 1–14 transmembrane regions. Some of those proteins are likely to be membrane transporters whose specificity could be tentatively predicted. One such protein, essential MMSYN1_0325, contains 16 helical regions, 12 of which are hydrophobic, likely transmembrane segments. Sequence database searches with the BLAST and HHPred programs show significant sequence similarity between MMSYN1_0325 and permeases of the major facilitator superfamily. Among the high-scoring matches (*E*-values between 10^−20^ and 10^−22^), there are transporter-like proteins with known spatial structure but unknown specificity and also several verified nucleoside and sugar transporters, including galactoside permease LacY (PDB 6VBG) and nucleoside transporter NupG (PDB 7DL9), both from *Escherichia coli*. There are also matches to sequence models for glucose transporters (NCBI CDD cd17315) and mellibiose symporters (NCBI CDD cd17332). The alignment of MMSYN1_0325 and LacY is shown in Fig. S1a. Taking a cue from the reconstruction of JCVI-Syn3.0 metabolism (12, 13), we speculate that MMSYN1_0325 could be the transporter for a sugar or sugar derivative, such as sialic acid or related N-acetylmannosamine. The operon for metabolizing these sugars is part of JCVI-Syn3.0, but the cognate transporter has not been accounted for. Alternatively, MMSYN1_0325 could facilitate the transport of nucleotides or nucleobases, another uptake capability that has been shown experimentally, but the transporter has not been identified. The experiments to identify those transporters were proposed as an immediate priority by Brewer et al. in 2019 (see Table 8 in reference 12).

The essential gene product MMSYN1_0691 is a protein with 14 transmembrane segments, showing significant sequence and structural similarity to many proteins from gram-positive and gram-negative bacteria. Some of the highest-scoring matches are annotated as members of “uncharacterized subfamily of the multidrug and toxic compound extrusion (MATE) proteins” (e.g., NCBI cd13139, HHPred *E*-value 10^−19^). Among the proteins whose function is inferred more specifically, the top matches are lipid flippase homologs from *E. coli* and other proteobacteria (PDB 7WAX, 9G97, and 6CC4; *E*-values 10^−17^–10^−18^). This is a group of ATP-independent lipid transporters that move phospholipids from the inner to the outer leaflet of the outer cell membrane. The best-studied representative of this family of flippases is the *E. coli* protein MurJ, known for its role in the final deposition of lipid II, the key building block of the cell wall peptidoglycan (33). Mycoplasmas lack cell walls, and their cell membrane is a single phospholipid bilayer; the phospholipid content may be asymmetric between the inner and outer leaflets, as suggested by the requirement of cholesterol, a known asymmetry factor, for mycoplasma growth (34). Interestingly, Hutchison et al. (11) suggested a flippase function for the heterodimer of ATPase MMSYN1_371 and membrane channel MMSYN1_372, both encoded by essential genes. The ATP-dependent flippase subunits from different proteobacteria are indeed among the highest-scoring matches to MMSYN1_371 and MMSYN1_372. It is not known why a minimal cell would require both ATP-dependent and ATP-independent flippase homologs. It is worth investigating whether MMSYN1_0691 or the MMSYN1_371/MMSYN1_372 pair, or all of them, play essential roles in phospholipid sorting in JCVI-Syn3.0.

Another essential protein with a putative phospholipid connection is the product of the essential gene MMSYN1_0249. This protein contains an N-terminal transmembrane segment, predicted to anchor the protein on the cytoplasmic side of the plasma membrane. The remaining sequence region appears to be distantly related to protein domains that belong to the pleckstrin homology-like (PH) beta-barrel fold. The highest-scoring HHPred match for MMSYN1_0249 in the PDB, to the PH domain of the human vacuolar protein sorting protein 36 (VPS36; PDB 2HTH), has an unimpressive *E*-value of 0.1, but direct structural matching to the PDB with the Dali program shows highly significant matches to 2HTH and many other PH domains. There are more than 20 structures with *z*-scores above 9 and hundreds of structures with *z*-scores above 5, all considered to be indicative of evolutionary relationship. A structure-based juxtaposition of the sequences of MMSYN1_0249 and VPS36 is shown in Fig. S1b. PH-fold domains are widespread in eukaryotes, occurring in many signaling and structural proteins; versions of the fold are also known as GRAM, GLUE, and FERM-C domains (tree node ID 220.1.1.217 in the ECOD database) (35). Most PH-related domains have the ability to bind anionic phospholipids, specifically phosphatidylinositols (36). Little is known about the prevalence of PH domains in bacteria, but it has been shown that mycoplasma growth requires fatty acids in culture medium and that two varieties of phospholipids are required to maintain the growth of a minimal mycoplasma cell (34). MMSYN1_0249 may play a role in phospholipid utilization by the minimal genome, possibly together with its genomic neighbors MMSYN1_0248 and MMSYN1_0250, which are both essential and completely uncharacterized.

One more membrane protein, MMSYN1_0332, contains three predicted transmembrane helices, two cytoplasmic regions, and one short external loop. The longest intracellular hydrophilic region of approximately 100 amino acids is predicted to be structurally similar to Zn-dependent metallopeptidases, particularly to a minimal version of those enzymes from the recently characterized M48 family (37). The highest similarity in the Dali search was to a putative metallopeptidase from *Acidothermus cellulolyticus* 11B (PDB 3E11, *z*-score 6.6). Among other significant matches, there was a membrane protein, jannalysin, which is a structurally dissected mini-protease from *Methanocaldococcus jannaschii* (PDB 4JIX, *z*-score 5.5). The enzymatic activity in many families of metallopeptidases depends on the sequence motif HExxH, in which two histidine residues bind a nucleophilic zinc ion, and the aspartate residue serves as a general acid (38). In MMSYN1_0332, the entire signature is replaced (Fig. S1c); hence, it is unlikely to be an active enzyme. However, the gene has been categorized as quasi-essential in JCVI-Syn3.0 (12). Interestingly, another high-scoring match of MMSYN1_0332 is to a region within a multidomain transcriptional regulator PrpR from *Mycobacterium tuberculosis* (PDB 6CZ6; *z*-score 6.2), in which the HExxH motif is also inactivated; MMSYN1_0332, however, has no regions of sequence similarity to known DNA-binding domains. The function of MMSYN1_0332 in the cell membrane remains to be understood.

Other proteins in Set C that contain transmembrane helices include the following. MMSYN1_0143 has eight transmembrane helices, predicted by AlphaFold to form two four-helix bundles. MMSYN1_0411 includes five transmembrane helices and a hydrophilic C-terminal region showing statistically significant similarity in HHPred search to the uncharacterized C-terminal domain of *Bacillus subtilis* YitT protein (*E*-values 10^−14^–10^−12^) and significant structural similarity in Dali searches to proteins with the GlnB fold, with almost 600 PDB structures with *z*-scores between 6 and 10 led by nearly 30 structures of representatives of nitrogen regulatory protein P-II family; this protein has been re-classified as non-essential in a previous study (12). MMSYN1_0296 and MMSYN1_0478 each have four transmembrane helices. MMSYN1_0601 has four transmembrane helices and a helical hydrophilic extension, and four proteins, MMSYN1_0235, MMSYN1_0778, MMSYN1_0830, and MMSYN1_0852, each consist of one transmembrane alpha-hairpin of two helices. The molecular functions of all those proteins remain unknown.

Essential MMSYN1_0338 is a putative lipoprotein that has no transmembrane segments but is likely to be conjugated to a membrane lipid through the cysteine residue in the predicted lipobox Val-Ile-Ala-Cys (23). AlphaFold indicates that the mature protein consists of two globular domains, each about 100 amino acids long. The N-terminal domain is matched by Dali to the Rib domain of *Lactobacillus acidophilus* (PDB 6SX1, *z*-score 5.1; Fig. S1d). Rib domains are also found in the surface proteins of streptococci and some other bacterial pathogens of animals. They are simplified versions of immunoglobulin-type beta-sandwiches, in which a pair of beta-strands on one edge is replaced by a helix (39). The second, C-terminal domain of MMSYN1_0338 also shows a Dali match, in this case with borderline statistical significance, to another immunoglobulin-like fold protein from bacteria, that is, the C-terminal domain in *Bacillus anthracis* S-layer protein Sap (PDB 6QX4, *z*-score 3.8). (Fig. S1d). In *B. anthracis*, disruption of Sap proteins causes severe defects in cell morphology and impaired growth in culture (40). Thus, MMSYN1_0338 is expected to reside on the outer surface of the membrane, perhaps utilizing the binding affinities of its immunoglobulin-like domains to preserve integrity and basic cell division functionality in the minimal cell.

Finally, quasi-essential MMSYN1_0392 is a protein of 100 amino acids. AlphaFold2 predicts a single three-stranded beta-sheet with two alpha-helices packed against one face. There are no transmembrane segments in MMSYN1_0392, nor does it show strong similarity to sequence databases. However, Dali search identifies significant matches to the C-terminal non-catalytic domains of the acyl-transfer enzymes (PDB 5D6J, *z*-score 8.4) and to cytoplasmic domains of the magnetosome subunits MamM (PDB 6HAN, *z*-score 7.6) (Fig. S1e). These domains are known to share the alpha-lytic protease prodomain-like fold (tree node ID 327 in the ECOD database) and are involved in interactions with small molecules. Based on a high structural similarity to the acyl-transfer auxiliary domain, which specifically binds ATP (41), we suggest that MMSYN1_0392 mediates acquisition or intracellular shuttling of a small ligand, perhaps a nucleotide.

### Bacterial immunity: variations on the toxin-antitoxin theme

In recent decades, many bacterial and archaeal genomes have been shown to harbor diverse two-gene modules that share a “self-addiction” property—a daughter cell that does not inherit the module either dies or cannot divide further. Some of these modules are involved in the maintenance of conjugative plasmids, in phage resistance, in bacterial persistence, or in cell recovery from stress (42–44). All such systems share certain basic principles of organization. A module usually includes a physically stable effector targeting an essential cell function and a less stable antagonist. The effector (toxin) and its antagonist (antitoxin) are typically encoded next to each other in the genome and are expressed together. The known toxins are mostly proteins, although sometimes they are non-coding RNAs; antitoxins also may be RNAs or proteins. The molecular mechanisms of toxin action are various, although DNase and RNase activities are common among them. Antitoxins act at different levels, including interference with the translation of the toxin gene, inhibition of toxin activity, or physical destruction of the toxin. Toxin-antitoxin (TA) systems are currently classified into eight types according to the chemical identity of antitoxins and their inhibitory mechanism (45).

Analysis of MMSYN1_0730, a quasi-essential gene product in JCVI-Syn3.0, reveals that this protein shares global sequence and structural similarity to the antitoxin class represented by the AbiEi protein from *Streptococcus agalactiae* (PDB 6Y8Q, HHPred *E*-value 2.3 · 10^−17^). The N-terminal DNA-binding domain of AbiEi binds to a genomic site upstream of the Abi operon, leading to inhibition of the expression of the TA pair *in vivo* (46). The C-terminal domain of AbiEi has a nucleotidyl transferase fold and prevents cell division arrest induced by the cognate toxin AbiEii. The toxin AbiEii is itself a nucleotidyl transferase, with two aspartate residues in a DxD signature; this sequence motif is essential for toxin activity (47). Interestingly, in several species, including *M. tuberculosis*, the antitoxin domains of AbiEi are mutated and the signature DxD is substituted by uncharged residues; hence, the antitoxin is unlikely to be an active enzyme. The entire domain structure and the inactivated DxD signature are conserved in the AbiEi homolog MMSYN1_0730. The adjoining MMSYN1_0731, a non-essential gene, is an AbiEii toxin homolog. The MMSYN1_0730/MMSYN1_0731 pair in *M. mycoides* has been included in specialized toxin-antitoxin databases PADLOC (48) and TADB 3.0 (49). Interestingly, PADLOC lists three AbiEi/AbiEii-related TA systems in *M. mycoides*, one of which appears to be pseudogenized (e.g., see entry GCF_001267815.1_ASM126781v1 in PADLOC); among all six genes, only MMSYN1_0730 is essential.

**Fig 4:**
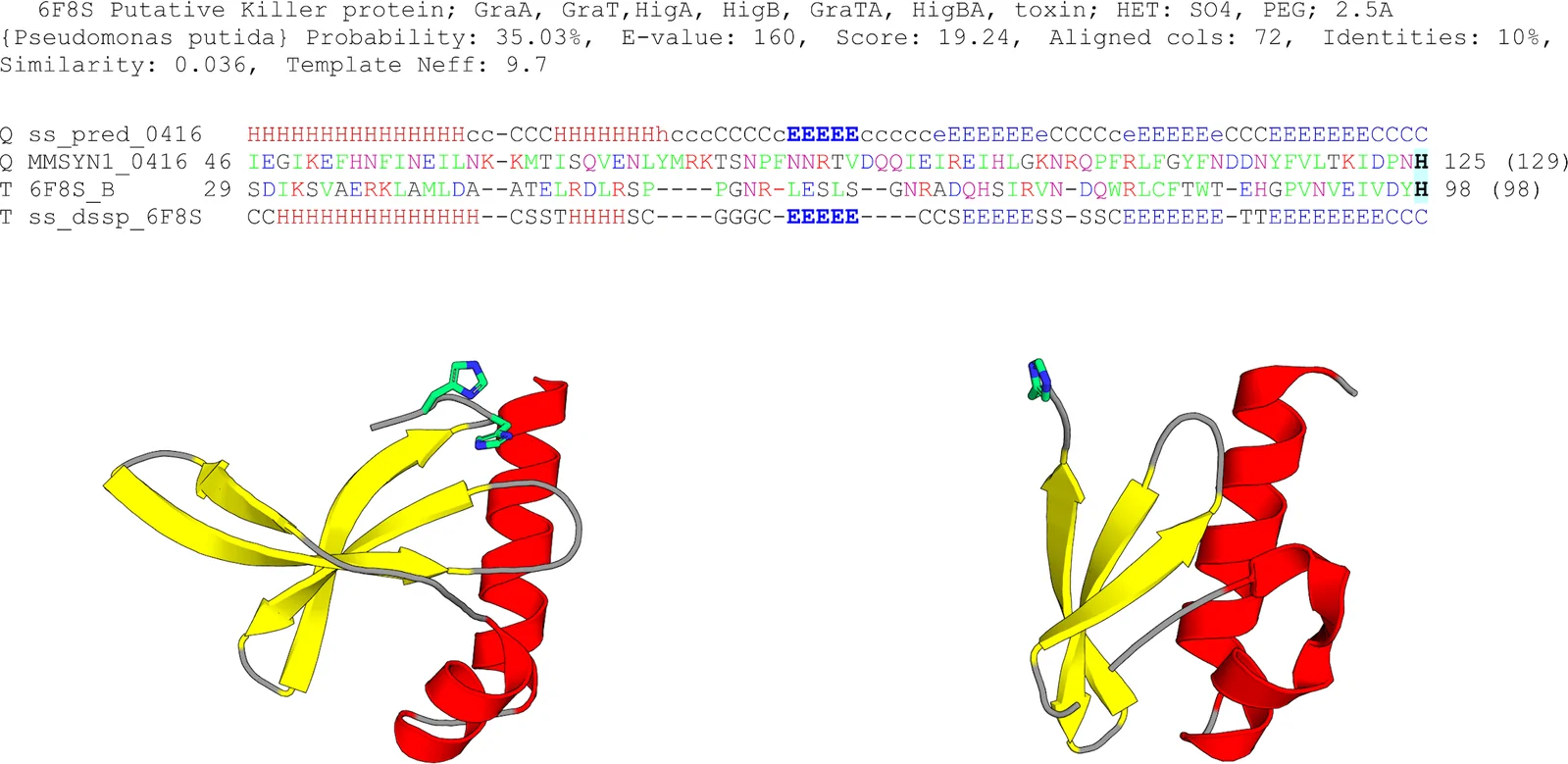
Structural similarity between RelE-family interferase GraT and MMSYN1_0146. Top, sequence match detected by the HHPred algorithm. In the secondary structure lines (ss), E stands for the extended structure (a beta-strand), and H stands for a helix. The bold type indicates a conserved strand that was not assigned by Dali but was identified by visual inspection of the PDB files. Bottom, spatial structures of MMSYN1_0146 (AlphaFold model, left) and GraT (PDB 6F8S, subunit B, right). The strand shown in bold type in the top panel is the closest to the viewer in MMSYN1_0146. In 6F8S, the corresponding region is rendered as a gray wire, but its extended structure is evident; the dipeptide GN following the strand is not resolved in 6F8S. The C-terminal histidine residues implicated in catalysis are rendered in neon green; MMSYN1_0146 has an extra histidine at the extreme C-terminus.

The protein encoded by the quasi-essential gene MMSYN1_0416 is a distant relative of RelE-like interferases, the RNA degradation enzymes with a unique spatial fold in the two-layer alpha + beta category (tree node ID 4312 “RelE-like” in the ECOD database). The HHPred search with the MMSYN1_0416 sequence model shows matches to several RelE homologs (such as *Pseudomonas putida* killer toxin GraT, PDB 6F8S), albeit without statistical significance, whereas Dali structural matching indicates an evolutionary relationship to RelE and many other interferases; for example, a match to *M. tuberculosis* toxin RelE-2, PDB 3G5O, had a suggestive *z*-score of 6.2 (Fig. 4). Neither HHPred nor Dali reported any matches to other protein families. The putative catalytic histidine residue located at the C-termini of RelE-family enzymes (50) is conserved in MMSYN1_0416, suggesting that it may be an active ribonuclease (Fig. 4). The beta-sheet that is found in the MMSYN1_0416 AlphaFold model has an added strand forming a beta-hairpin bent out of the main sheet at an angle, and inspection of the GraT structure 6F8S suggests a similar feature, not annotated as an extra strand in PDB (Fig. 4).

In the *M. mycoides* genome, MMSYN1_0416 is located next to a non-essential gene MMSYN1_0417, which encodes a member of an uncharacterized protein family DUF4065. The best database match for MMSYN1_0417 is a fragment of the DarG protein of *M. tuberculosis* (PDB 7YK3, marginally significant *E*-value 10^−3^), the antitoxin from a different TA pair. Antitoxin DarG consists of two domains: the N-terminal domain that belongs to the Macrodomain family with monoADP-ribose hydrolase activity and the C-terminal helical domain that can bind to and inhibit DarT toxin (51). MMSYN1_0417, as well as other DUF4065 family proteins, is homologous to the C-terminal helical antitoxin/inhibitor domain of DarG but lacks the Macrodomain. Thus, MMSYN1_0416 and MMSYN1_0417 appear to be a non-cognate toxin-antitoxin pair encoded in a putative operon. It is unknown whether MMSYN1_0417 could inhibit MMSYN1_0416 activity; this question awaits experimental resolution. In any case, it is curious that the MMSYN1_0416 gene essential for cell survival is a toxin homolog. Neither MMSYN1_0416 nor MMSYN1_0417 are captured in PADLOC or TADB 3.0.

**Fig 5:**
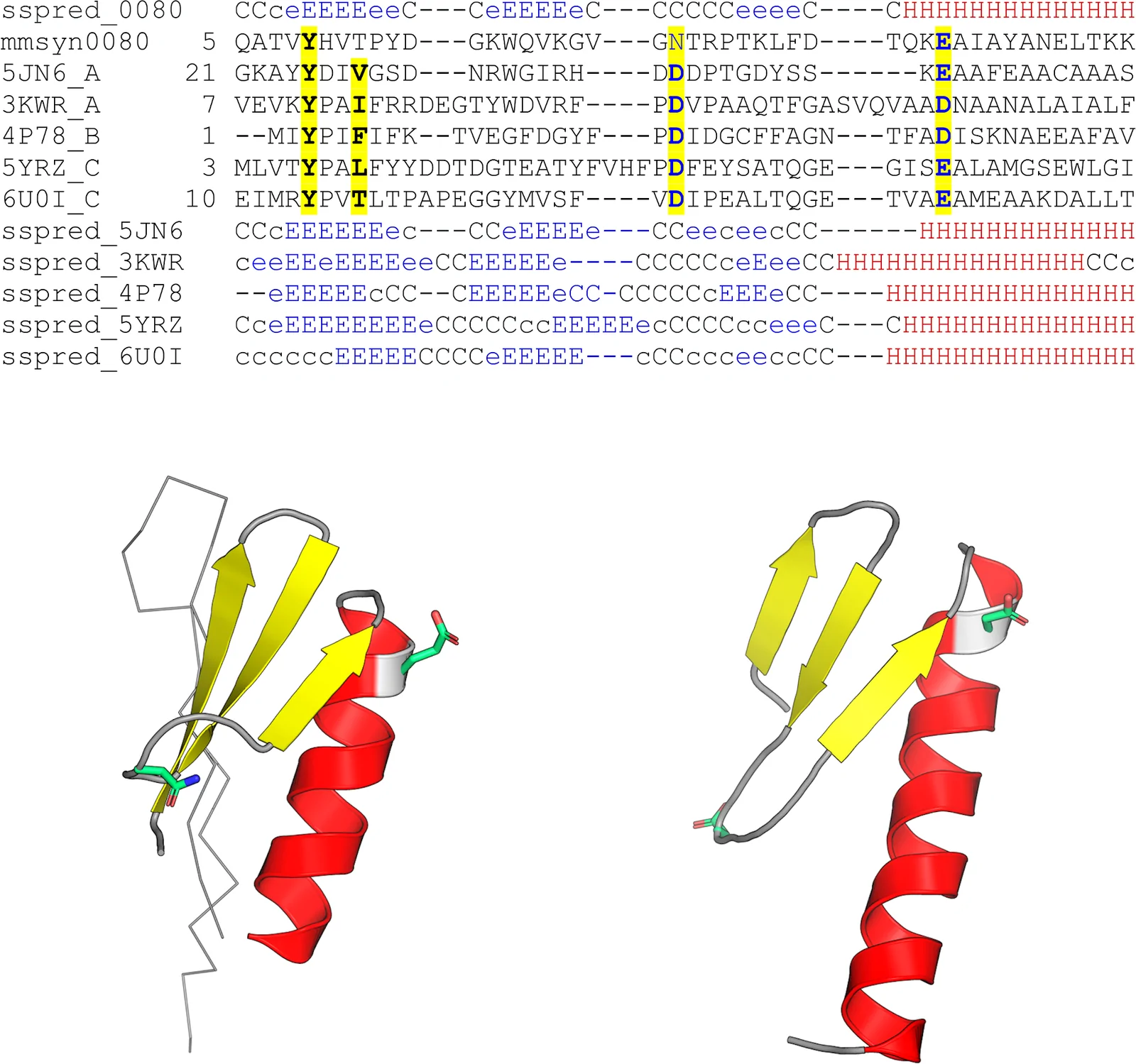
Sequence and structural similarity between MMSYN1_0080 and HicB antitoxins. Top, sequence similarities. A partial match was detected by the HHPred algorithm, and a fuller alignment was obtained by the MUSCLE program and rendered in the HHPred style. Bottom, structural similarity between the MMSYN1_0080 model (left) and the HicB3 antitoxin of *Yersinia pestis* 4P78 (right). The two conserved acidic residues discussed in the text and highlighted in the top panel are rendered in neon green. The two strands in MMSYN1_0080 that extend the sheet are not included in the sequence alignment and are shown as gray wires.

Three other Set C proteins, MMSYN1_0080, MMSYN1_0281, and MMSYN1_0805, present further variations on the theme of bacterial immunity factors. One of those, MMSYN1_0080, is assigned in the databases to the uncharacterized family PF09954/DUF2188. The HHPred search shows a single instance of high sequence similarity between MMSYN1_0080 and the RPA3313 protein from *Rhodopseudomonas palustris* (PDB 5JN6, *E*-value 2 · 10^−14^), followed by a suggestive similarity to a portion of a larger antitoxin HicB from *Streptococcus pneumoniae* (PDB 5YRZ, *E*-value 0.5). Despite the lack of statistical significance in the latter case, it is notable that in the iterative HHPred searches, MMSYN1_0080, 5JN6, and many recognized HicB homologs form a network of top-scoring matches (data not shown). The predicted structure of MMSYN1_0080 and the known HicB structures are very similar, consisting of a beta-sheet core with three antiparallel strands, followed by a helix packed against the side of the sheet (Fig. 5), sometimes extended by additional strands located C-terminally to the helix along the protein chain. This core has been recognized as a simplified RNaseH-like fold (52). A conserved acidic residue identified at the beginning of the helix is also seen in MMSYN1_0080, and we observed another highly conserved aspartic acid residue in the loop between strands 2 and 3 at the opposite tip of the molecule, replaced by an asparagine in MMSYN1_0080 (Fig. 5). The portion of HicB proteins that aligns with MMSYN1_0080 is in their N-terminal domains, which is sufficient to inhibit the activity of its cognate toxin, ribonuclease HicA (53, 54). The C-terminal domain of HicB involved in nucleic acid binding is not conserved in MMSYN1_0080. Thus, MMSYN1_0080 might be a minimal antitoxin module, but there is no suitable toxin homolog in its genomic neighborhood. A later round of mutagenesis suggested that MMSYN1_0080 is a non-essential gene (12), but the locus may in fact have multiple functions (see more on this in Discussion).

An essential gene MMSYN1_0281 is annotated as a conserved hypothetical protein in GenBank but is not assigned to any protein family. The HHPred search shows that MMSYN1_0281 is similar to mono-ADP phosphoribosyltransferases; coincidentally or not, the best match is to the DarT toxin discussed earlier in this section (PDB 8BAR, *E*-value 1.2 · 10^−4^). The predicted ligand-binding cleft is conserved in MMSYN1_0281, but the four residues essential for the activity (55)—that is, the nucleophilic Glu152, base-coordinating Asn104, intermediate-stabilizing Met71, and Arg52 that aids in proton abstraction—are all substituted in MMSYN1_0281 (Fig. S2a), suggesting that this essential gene encodes an inactivated enzyme. The neighborhood of MMSYN1_0281 does not include an obvious antitoxin.

An essential gene product MMSYN1_0805 belongs to yet another protein family that includes bacterial toxins, the vast Doc/Fic family of adenylyl monophosphate transferases (AMPylases). Doc/Fic AMPylases are broadly conserved in all domains of life and have a distinct all-alpha-helical fold not related to the structure of nucleotidyltransferases discussed earlier in this section. In bacteria, Doc/Fic-domain proteins are often found as the toxin components of TA systems or sometimes as standalone toxins. An example of the former is the Fic-domain protein of *Bartonella schoenbuchensis*, called VbhT, and its cognate antitoxin VbhA (56). MMSYN1_0805 is very similar to the N-terminal domain of VbhT (PDB 3SHG, HHPred *E*-value 2 · 10^−17^), but the C-terminal portion of MMSYN1_0805 does not align with VbhT. Moreover, there is no VbhA homolog in *M. mycoides*. An example of a stand-alone Doc/Fic toxin is the CccR protein in *Yersinia pseudotuberculosis*, which is a fusion of an AMPylase domain with the C-terminal helix-turn-helix DNA-binding module. MMSYN1_0805 is highly similar to CccR (PDB 7CB8, *E*-value 6 · 10^−32^) in both Doc/Fic and DNA-binding domains, but the catalytic histidine residue in the key Doc/Fic motif is replaced by a serine (Fig. S2b). Thus, it is unclear whether MMSYN1_0805 retains nucleotidyltransferase activity. Moreover, the CccR protein in *Y. pseudotuberculosis* is secreted by the type VI secretion system, which *M. mycoides* does not have; hence, the mode of action of the essential gene product MMSYN1_0805 in JCVI-Syn3.0 remains unknown.

### Further immunity connections: MMSYN1_0389 specifies a novel lineage of Cas4-related nucleases

Deoxyribonuclease Cas4 is best known as a member of the CRISPR-Cas system of innate immunity in prokaryotes. About 90% of archaeal genomes and more than 20% of bacterial genomes encode at least one Cas4 homolog (56). The *cas4* gene has more autonomy than many other members of the CRISPR-Cas system; it can be located within *cas* operons (“CAS-*cas4*”) but is also found in genomic locations far from any other *cas4* genes (“solo-*cas4*”) or sometimes in phage genomes or mobile genetic elements (57). Within *cas* operons, Cas4 proteins are part of the “adaptation module.” They enable acquisition of invading non-self DNA, processing it into fragments, and insertion of the fragments as spacers into CRISPR repeats. The Cas4 protein associates with the conserved nuclease/integrase complex Cas1/Cas2, binds to the protospacer adjacent motif (PAM) region, and cleaves the PAM precisely to leave the 3′ overhang and ensure the correct orientation of the spacer. The substrate processing and its integration in the genome is then performed by the Cas1/Cas2 complex; see previous studies (58–60) for many mechanistic details. In contrast, the biological roles of solo-Cas4 proteins are not well understood.

A product of the quasi-essential gene MMSYN1_0389 is annotated as a member of the PF11074/DUF2779 family of proteins, which are widely distributed in bacteria but not functionally characterized. The HHPred comparisons of the MMSYN1_0389 sequence family model with the databases suggest that this protein is a fusion of two distinct regions, both of which are likely to have exonuclease activity. The N-terminal region of MMSYN1_0389, approximately up to amino acid 380, is similar to Cas4 nucleases. The highest-scoring match to the Cas4 portion of the *Geobacter sulfurreducens* Cas4-Cas1 fusion protein (PDB 7MID) has an *E*-value of 2 · 10^−6^, and homologs from several other species show *E*-values between 10^−3^ and 10^−5^. In the following, we used a Cas4 homolog from *Sulfolobus sulfataricus* (PDB 4IC1) for comparison, as HHPred generated a fuller alignment of this protein with MMSYN1_0389.

The C-terminal region of MMSYN1_0389, starting approximately at residue 460, has similarity to a different family of DNA exonucleases and ribonucleases with a distinct full (non-degraded) RNase H fold, particularly to a lineage known as DEDD/DEDDh nucleases. The best-scoring match is to mitomycin C repair nuclease, a DEDDh nuclease from *B. subtilis* (PDB 8UN9; *E*-value, 5 · 10^−7^).

**Fig 6:**
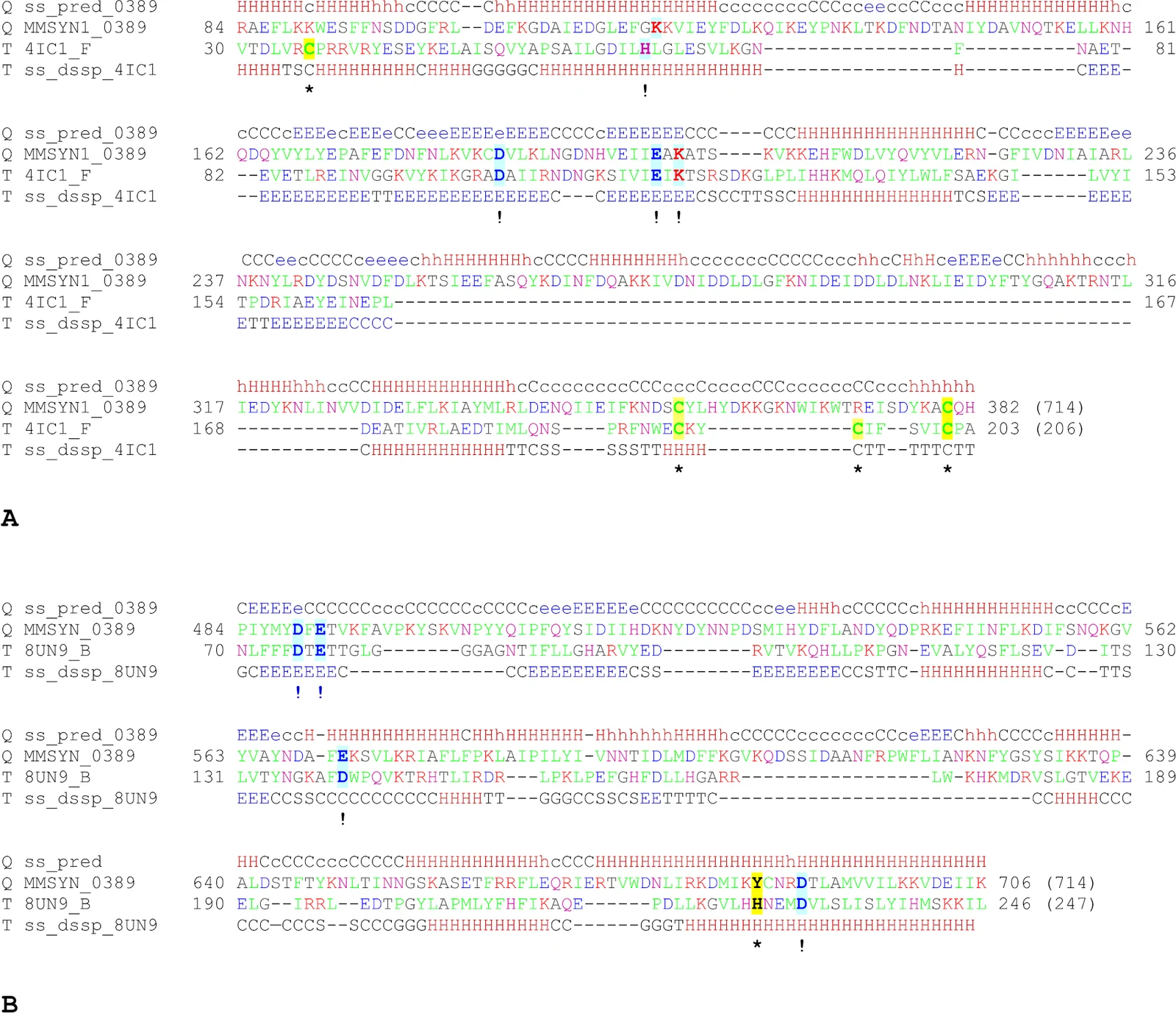
Two domains in MMSYN1_0389 are related to two different nucleases. (**A**) HHPred alignment between the N-terminal half of MMSYN1_0389 and a Cas4 homolog PDB 4IC1. In the secondary structure lines (ss), E stands for the extended structure (a beta-strand), and H stands for a helix. The bold type with light blue shading and the exclamation mark indicates the conserved charged residues playing direct roles in catalysis in Cas4, and yellow shading with asterisks below the alignment indicates the cysteine residues coordinating the Fe-S cluster in the known Cas4 but partially replaced in MMSYN1_0389. (**B**) HHPred alignment between the C-terminal half of MMSYN1_0389 and exonuclease MrfB, a DEDDh-class nuclease (PDB 8UN9). The bold type with light blue shading and the exclamation marks indicate the conserved set of acidic residues playing a direct role in catalysis, and yellow shading with an asterisk below the alignment indicates the histidine-to-tyrosine substitution.

Closer analysis of MMSYN1_0389 similarities suggested that both the N-terminal and the C-terminal domain are likely to be catalytically active nucleases (Fig. 6). The *Geobacter* and *Sulfolobus* Cas4-mediated DNA cleavage reaction depends on the added manganese *in vitro*, and structural analysis of 4IC1, supported by its comparison with 7MID and with homologous bacterial deoxyribonucleases AddA and RecB involved in DNA repair, shows that 4IC1 contains a bound metal ion (61). The ion is ligated by a partial positive charge from the side chain atom in His62 (His66 in the alignment), negative charges from the side chain atoms in Asp99 and Asp113, and partial charge from the main chain atom of Lys115. The latter three residues are conserved throughout the PF11074/DUF2779 family, represented in MMSYN1_0389 by Asp186, Glu210, and Lys212, respectively (Fig. 6A). The upstream histidine in MMSYN1_0389 is aligned to a glycine, followed by lysine, whose long side chain could provide the remaining partial positive charge. Interestingly, another feature of most Cas4 proteins, that is, four cysteine residues that can chelate a Fe-S cluster, is not preserved in MMSYN1_0389, where only two cysteines are found (Fig. 6A). In *S. sulfataricus*, the Fe-S cluster bound by cysteines appears to be essential for catalysis, but in a recently characterized solo-Cas4 from *Methanosarcina mazei,* the mutant that no longer retains the Fe-S cluster remained catalytically active (62).

The C-terminal, DEDD-like domain of MMSYN1_0389 is similarly expected to be an active enzyme. The name of the family is derived from the four highly conserved acidic residues ligating two divalent (most often Mg^2+^) cations in the catalytic center. All four of those acidic residues are conserved in MMSYN1_0389. In a specific subset of DEDD-like nucleases, known as DEDDh, the fifth conserved residue, a histidine (“h” in the name), is located 4–5 residues before the last aspartate; this histidine, sometimes substituted by tyrosine, may polarize a water molecule, which would act as a nucleophile initiating the cleavage reaction (63). In MMSYN1_0389, a tyrosine is found in the right position (Fig. 6B).

### Other essential genes: inactivated versions of three enzymes and a “complete unknown” with an interesting structure

We identified interesting features in several other proteins. One of them is the quasi-essential MMSYN1_0777, a protein with a long (36–37 amino acids) N-terminal hydrophobic segment, likely attaching the protein to the cell membrane. The hydrophilic part of MMSYN1_0777 shows an HHPred match to the “GIY-YIG” family of endonucleases, so called after the namesake conserved sequence signature. The GIY-YIG enzymes include a class of bacterial restriction endonucleases, a subset of Holliday junction resolvases in eukaryotes, and widely distributed mobile intron-encoded homing endonucleases (64, 65). The similarity between MMSYN1_0777 and the GIY-YIG enzymes is not statistically significant (*E*-value 60 for the best match, PDB 3MX1), but the conservation of all elements of the secondary structure is notable. Several of the residues essential for enzymatic activity are, however, replaced in MMSYN1_0777, including one of the two tyrosines interacting with the nucleophilic water molecule and the proton-abstracting histidine (65). Thus, the quasi-essential protein MMSYN1_0777 may be an inactivated copy of a GIY-YIG endonuclease (Fig. S3a).

Quasi-essential MMSYN1_0138, according to the AlphaFold prediction, has a three-domain structure. The first domain, composed of two discontinuous segments of the chain (amino acids 1–95 and 385–510), has an alpha-beta Rossmann-like fold. The sequence of this domain is interrupted by an insert that folds into the second, alpha-helical domain without disturbing the Rossmann-like domain. The structure is completed by the third, C-terminal domain (amino acids 515–630), folded as two small beta-barrels. The HHPred analysis of the spliced sequence of amino acids 1–95 and 385–510 strongly suggests that MMSYN1_0138 is homologous to an ATP-dependent transporter (the highest-scoring full-length match, the cell division ATPase FtsE of *M. tuberculosis*, PDB 8IGQ, has the *E*-value of 2 · 10^−9^). However, the Walker A and Walker B motifs, which are essential for catalysis, are mutated in MMSYN1_0138, suggesting that it is not an active ATPase.

MMSYN1_0505, a predicted membrane-anchored lipoprotein, shows structural similarity, in a Dali search, to thioredoxin-like and glutaredoxin-like proteins. The database matches extend along the full lengths of thioredoxins; the highest *z*-score of 8.5 is to *Caulobacter crescentus* thioredoxin Trx1, and there are dozens of other matches with *z*-scores greater than 7. This suggests a high structural similarity and possibly an evolutionary relationship between thioredoxins and MMSYN1_0505. That said, the hallmark of functional thioredoxins and their relatives is a signature of two cysteines, arranged as a CxxC pair (most commonly WCGPC) and involved in the reduction/oxidation of sulfhydryl groups (66). This signature, including both cysteines, is mutated in MMSYN1_0505. Thus, MMSYN1_0505 has a thioredoxin fold but is unlikely to be a functional thioredoxin; it has been reclassified as non-essential after an additional round of mutagenesis in JCVI-Syn3A (12).

**Fig 7:**
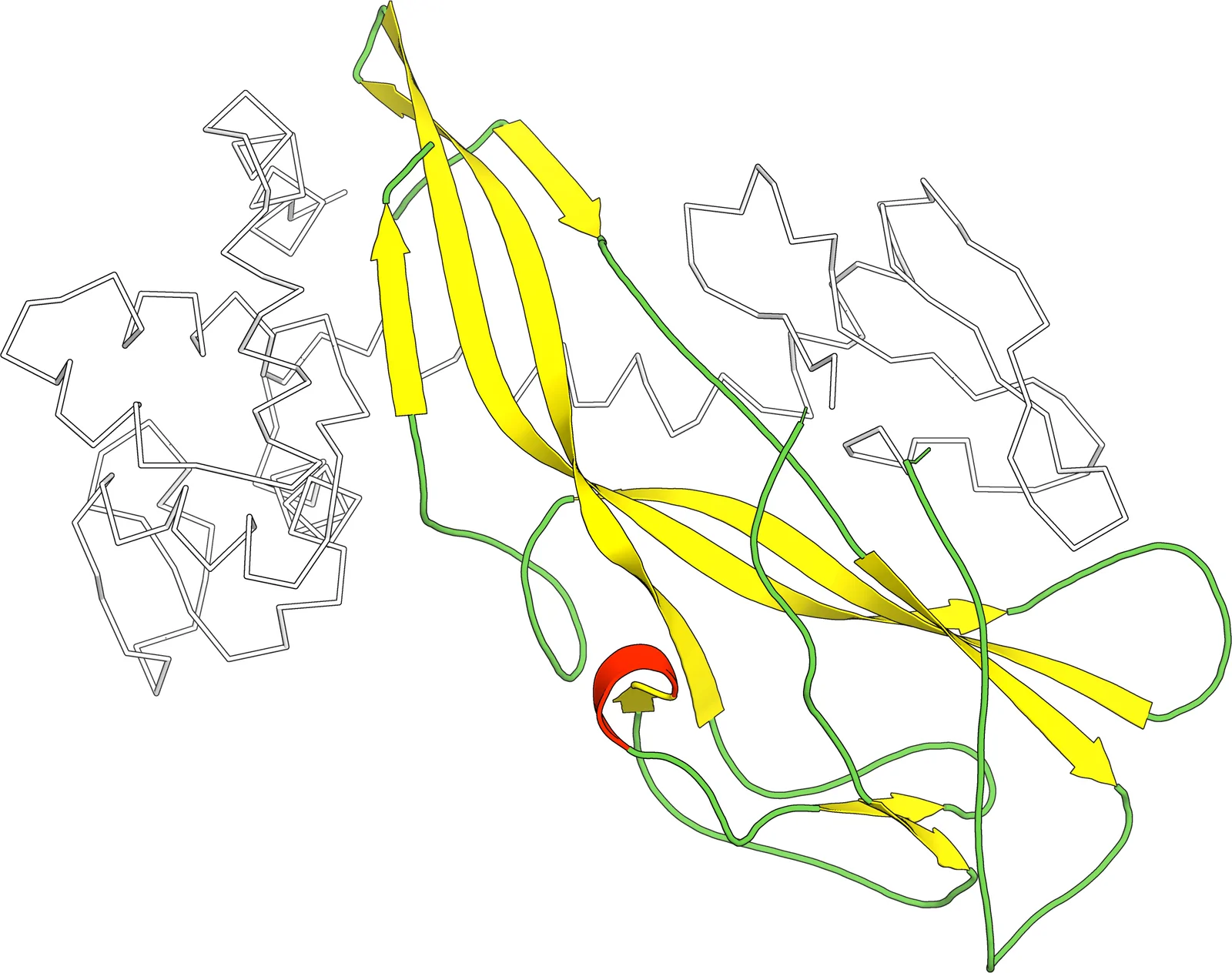
MMSYN1_0835 has a complex, likely novel, fold. The confidently predicted C-terminal all-beta domain is rendered as colored ribbons. The N-terminal domain, predicted less confidently, is rendered in white wires, and the leader peptide is not shown.

Finally, MMSYN1_0835, a 435 amino acids-long protein, contains a putative leader peptide, likely mediating its export from the cell. The AlphaFold model of MMSYN1_0835 consists of two regions. The N-terminal half of the protein is a collection of helices, strands, and loops that are not well packed and are predicted with low confidence. In contrast, the C-terminal portion of the protein, starting approximately at amino acid 200, is confidently predicted to form a discrete domain with a peculiar fold. It is dominated by two long beta-strands, bent in half nearly at the right angle, with several shorter strands added to each half. The overall arrangement, supplemented by a few helices, resembles a cupped palm (Fig. 7). We could not detect significant sequence or structure similarities to either of the two domains of MMSYN1_0835 in PSIBLAST, HHPred, Dali, or FoldSeek searches of public sequence and structure databases. The general shape of the C-terminal domain is reminiscent of ribonucleases with the RNase A fold, which feature a similarly bent, or bilobed, beta-sheet (67). However, the dimensions of MMSYN1_0835 and its strand connectivity are different. The essential function of MMSYN1_0835 awaits experimental determination. This is more an unsolved puzzle than a functional prediction, and MMSYN1_0835 is not included in Table 3.

**TABLE 3:** Summary of the novel predictions in this work^*a*^

Similarity and putative function	Protein ID	Sorting signals	Match in PDB	Algorithm	Essentiality	PROST
Membrane-related functions						
Sugar permease	MMSYN1_0325	NTM	6VBG	HHPred	Essential	**Niacin/nicotinamide transporter**
N-domain, putative permease; C-domain, GlnB-like	MMSYN1_0411		3HLU	N-domain, HHPred, C-domain, AF + Dali	Non-essential	**Yop translocation protein U** (N-domain)
Flippase	MMSYN1_0691	NTM	6CC4	HHPred	Essential	**Flippase MurJ**
Pleckstrin homology domain, phospholipid-binding	MMSYN1_0249	NTM	8UJW	HHPred	Essential	**GLABRA2 expression modulator** (lipid-binding START domain)
Inactive membrane-bound metalloprotease	MMSYN1_0332	NTM	3E11	AF + Dali	Quasi	**Putative membrane protease YugP**
Membrane-tethered protein, two Ig-like domains	MMSYN1_0338	LP	6SX1	AF + Dali	Essential	**Antigenic membrane protein**
			6QX4	AF + Dali		
Acyl transfer-like domain	MMSYN1_0392		5D6J	AF + Dali	Quasi	N/A
Immunity-related functions						
AbiEi antitoxin; polar in MMSYN1_0728	MMSYN1_0730		6Y8Q	HHPred	Quasi	N/A
RelE toxin; polar in MMSYN1_0415	MMSYN1_0416		4ML2**,** 6F8S	HHPred	Quasi	**Toxin RelG**
HicB antitoxin; polar	MMSYN1_0080		5YRZ	HHPred	Non-essential	Methane monooxygenase component D
Inactive DarT toxin	MMSYN1_0281		8BAR	HHPred	Essential	N/A
Inactive Doc/Fic AMPylase toxin; polar	MMSYN1_0805		3SHG	HHPred	Essential	**Protein adenylyltransferase SoFic**
Cas4-like nuclease fused to DEDD nuclease	MMSYN1_0389		7MID**,** 8UN9	HHPred	Quasi	N/A
Other						
Inactive GIY-YIG-family nuclease	MMSYN1_0777	NTM	3MH1	HHPred	Quasi	KilB plasmid transfer protein
Inactive Rossmann-fold ATPase	MMSYN1_0138		8IGQ	HHPred	Quasi	**Spermidine/putrescine import ATP-binding protein PotA**
Thioredoxin-like fold	MMSYN1_0505	NTM	3P2A	AF + Dali	Non-essential	N/A

^*a*^ In the Algorithm column, AF stands for AlphaFold 2-Monomer method. In the Sorting signals column, NTM stands for N-terminal transmembrane helix, and LP stands for leader peptide. The PROST predictions (68) are shown for comparison; those consistent with our results are highlighted in bold type. N/A indicates that PROST did not make a functional prediction for this protein.

## DISCUSSION

In this work, we identified sequence and structural similarities for 16 uncharacterized proteins encoded by the minimal synthetic genome JCVI-Syn3.0. These predictions and observations are summarized in Table 3.

Our analysis shows that the sequences of the least-understood putative proteins encoded in the JCVI-Syn3.0 genome (“Set C” in this study) tend to be shorter, enriched in alpha-helical regions, and include fewer annotated sequence domains than the JCVI-Syn3.0 genome and the entire *M. mycoides* genome. However, in many respects, the proteins in Set C are rather garden-variety bacterial proteins; they span a range of sizes, are not particularly disordered, and display diverse domain compositions and structural topologies. Furthermore, nearly one-third of all proteins in Set C contain conserved sequence signatures and structural similarities to protein families in the databases. The ongoing updates in the collections of conserved sequences and structures, as well as the improved sensitivity and selectivity of the search algorithms, undoubtedly all played a role in this latest gain of information.

The newly identified functions of Set C proteins mostly fall into two categories, that is, proteins likely to be involved in the transport of (small) molecules across the membrane or in membrane maintenance, and proteins whose homologs in other species are involved in managing genetic conflicts between bacteria and other organisms. Proteins in the second category are particularly interesting.

Generally speaking, a deletion of an antitoxin gene would be expected to have a lethal phenotype, as its cognate toxin would then be activated. In contrast, a deletion of a toxin typically should not affect cell survival in a rich medium, as the remaining antitoxin might not have its own phenotype in the absence of the toxin and, in any case, is likely to be unstable. This expectation is confirmed in the case of the MMSYN1_0730-MMSYN1_0731 pair: the product of the MMSYN1_0730 gene, the AbiEi-like antitoxin, is essential in single-gene deletion experiments, most likely because deletion de-inhibits the AbiEii-like toxin MMSYN1_0731, which in itself is dispensable and is not part of JCVI-Syn3.0. It could be expected that in JCVI-Syn3.0, antitoxin MMSYN1_0730 would no longer be essential, as toxin MMSYN1_0731 has already been deleted. The repeated transposon mutagenesis in the JCVI-Syn3.0 genetic background, however, shows that MMSYN1_0730 is quasi-essential (12), suggesting that it may be a multifunctional or perhaps even moonlighting protein, performing functions beyond an antitoxin role.

The naive logic appears to be further subverted in the cases of other toxin-like and antitoxin-like genes in JCVI-Syn3.0. The MMSYN1_0416-MMSYN1_0417 pair includes a toxin and an antitoxin, but the two proteins are not cognate; we are not aware of the cases in which RelE toxins are paired with DarG antitoxins. Moreover, the essentiality of the genes is reversed in this case, that is, the putative toxin gene MMSYN1_0416 is quasi-essential, while its adjoining antitoxin gene MMSYN1_0417 is not essential. Further puzzles are two toxins, DarT-like MMSYN1_0281 and Doc-like MMSYN1_0805, both of which are essential genes that encode apparently catalytically inactive enzyme derivatives. Finally, there is an essential Cas4 homolog MMSYN1_0389, encoding a fusion of two nucleases, both of which are likely to be active enzymes.

Why would homologs of putative toxins and immunity factors be necessary for the growth of a small bacterium on a parasite-free, nutrient-rich medium, and how are their essential or quasi-essential roles performed by apparently inactivated enzymes? One resolution for both of these problems could be that these loci are essential not because of the proteins they encode but because they exhibit polar effects on neighboring genes. This has been studied during the construction of JCVI-Syn3.0 by identifying transposon insertants whose survival depended on the orientation of the insert (69). That work discovered polar insertions in the open reading frames of MMSYN1_0080 and MMSYN1_0805 and also in the genomic regions located one or two genes apart from MMSYN1_0416 and MMSYN1_0730. Even so, this does not explain the lethal deletions of MMSYN1_0281 and MMSYN1_0389; these genes must encode essential products that play a role beyond bacterial immunity. We also cannot exclude the possibility that some of the putatively essential open reading frames in JCVI-Syn3.0 contain functionally important overlapping DNA or RNA elements, although there is no evidence for this thus far.

Since the 2016 study by Danchin and Fang (16) that we used as a starting point for collecting the unannotated proteins in JCVI-Syn3.0, there have been various efforts aimed at predicting molecular functions across the entire JCVI-Syn3.0 gene set (12, 13, 18, 68, 70–72) or specifically inferring the toxin-antitoxin systems in *M. mycoides* (73). The comparison of these predictions with our work is shown in Table S2. It is notable that only two membrane transporters (MMSYN1_0038 and MMSYN1_0325) and one potential immunity protein (MMSYN1_0805) have been annotated by more than one of those efforts, and apparently, none of these works noticed the inactivating mutations in MMSYN1_0038 and MMSYN1_0805. The most successful prediction was a study that used PROST, a consensus of several methods of structure and sequence similarity analysis (74). PROST detected about half of the similarities that we report here, mainly in the membrane proteins group; their functional interpretations of many of these similarities, however, are different from ours (Table 3; Table S2).

The ultimate definition of the biological functions of Set C proteins requires further experimentation. The molecular functions that are likely to be operational in JCVI-Syn3.0 but that lack an assigned dedicated gene product have been listed in Table 8 of reference 12, and the observations reported here may be used to test some of the plausible candidates.

Our study also illustrates the role that AlphaFold models play in functional annotation of genomic information. From the text and Table 3, it can be seen that more than half of our inferences of protein function required only comparisons of sequence family models to the protein families database; the gain in knowledge did not depend critically on the three-dimensional structure models generated by AlphaFold. However, even in such cases, AlphaFold models are useful, as they help dissect proteins into discrete structural domains, and sequences of these domains tended to be more convenient queries in database searches than entire multidomain proteins. However, sometimes, sequence-level analysis was inconclusive, whereas Dali or FoldSeek searches of the PDB with AlphaFold models proved to be informative. In the foreseeable future, we may expect that *de novo* structure prediction and comparison to sequence domain databases will continue to play complementary roles in predicting protein functions and dissecting cellular pathways.

## Data Availability

All data are publicly available, with their accession numbers provided in the article or the supplemental material.

## SUPPLEMENTAL MATERIAL

Figure S1 msphere.00077-26-s0001.pdf Sequence similarities in membrane-associated proteins of JCVI-Syn3.0.

Figure S2 msphere.00077-26-s0002.pdf Sequence similarities in immunity-associated proteins of JCVI-Syn3.0.

Figure S3 msphere.00077-26-s0003.pdf Sequence similarities in GIY-YIG endonuclease-like protein in JCVI-Syn3.0.

Supplemental legends msphere.00077-26-s0004.pdf Legends to supplemental figures and tables.

Table S1 msphere.00077-26-s0005.xlsx Protein IDs and sequences of the set C proteins.

Table S2 msphere.00077-26-s0006.xlsx Comparison of functional annotations made in several studies.

ASM does not own the copyrights to Supplemental Material that may be linked to, or accessed through, an article. The authors have granted ASM a non-exclusive, world-wide license to publish the Supplemental Material files. Please contact the corresponding author directly for reuse.
