# Supplementary material for: Minimal organism JCVI-syn3.0, an engineered derivative of Mycoplasma mycoides, encodes an assortment of unrecognized membrane transporters and immunity factors
Source: mSphere. 2026 Aug 28;11(9):e00077-26. doi: 10.1128/msphere.00077-26 (PMC13621843; doi:10.1128/msphere.00077-26)
Supplement: Supplementary file 1 [file msphere.00077-26-s0001.pdf]

```
6VBG_A Galactoside permease; HET: GAL, YIO, BNG; 2.8A {Escherichia coli} SCOP: f.38.1.2 Probability: 99.93%,  E-value: 1e-21,  Score: 199.3,
Aligned cols: 403,  Identities: 12%,  Similarity: 0.01

  Q ss_pred_0325      HHhcCChhHHHHHHHHHHHHHHHHHHHHHHHhhcchhHHhh-CCcHHHHHHHHHHHHHHHHHHHHHHHHHHHHHhccchH
  Q MMSYN1_0325  26  YKNSISKQKGLFYLEISSFWIVINFLIQIITNYIDSPILKS-FSSSTLTILLFLSSYFLYATILNPFALWLTLKLQSRRI 104
  T 6VBG          1  MYYLKNTNFWMFGLFFFF-----YFFIMGAYFPFFPIWLHDINHISKSDTWIIFAAISLFSLLFQPLFGLLSDKLGLRKY 75
  T ss_dssp_6VBG     CCCTTCHHHHHHHHHHHH-----HHHHHHHHSTTHHHCCCCCSCCCSTTHHHHHHHHHHHHHHHHHHHHHHHHHHTTSCH

  Q ss_pred_0325      HHHHHHHHHHHHHHHHH--HCCCCHHHHHHHHHHHHHHHHHHHHHHHHHHHhhcccc--cccchHHHHHHHHHHHHHHHHH
  Q MMSYN1_0325 105  WIWISLFSCFLSVMIAF--LSNVNITSIIFISLFLAVGISAQIIYFLFFNEQFNER--LFPVFSSIKAGFVISFATFISY 180
  T 6VBG_A       76  LLWIITGMLVMFAPFFIFIFGPLLQYNILVGSIVGGIYLGFCFNAGAPAVEAFIEKVSRRSNFEFGRARMFGCVGWALCA 155
  T ss_dssp_6VBG     HHHHHHHHHTTHHHHCCCCCHHHHHHCHHHHHHHHHHHHCCCCCTHHHHHHHHHHHHHHHTTCCHHHHHHHHHHHHHHHH

  Q ss_pred_0325      HHHHHHHHHhccCCChhHHHHHHHHHHHHHHHHHHHHHHccccccc--------cccccHHHHHHhhhhccHHHHHHHHH
  Q MMSYN1_0325 181  EVYSLLNLNLISNHNNYTNWIIFSLSLVCLIICLVVSIFVKERKIK--------VIKYKEDIVEQLQRYGYKVLIGLIVM 252
  T 6VBG_A      156  SIVGIMFTI--------NNQFVFWLGSGCALILAVLLFFAKTDAPSSATVANAVGANHSAFSLKLALELFRQPKLWFLSL 227
  T ss_dssp_6VBG     HHHHHHTTT--------CSSHHHHHHHHHHHHHHHHHHHSCC-------------CCTTSCCHHHHHGGGGSHHHHHHHH

  Q ss_pred_0325      HHHHHHHHHHHcchHHHHHHHHHHHhcCCchhhHHHHHHHHHHHHHHHHHHHHHHHHHHHHHHHhHHHHHHHHHHHHHHH
  Q MMSYN1_0325 253  SFLITSVNVIIKSDIFELFLVSKLKQQSYTSLNVWNYLQSFRLSFVLGQLLLGYLFYKLVIKVIGIVKSISILTSLTMFG 332
  T 6VBG_A      228  YVIGVSCTYDVFDQQFANFFTSFFATGEQGTRVFWYVTTMGELLNASIMFFAP-----LIINRIGGKNALLLAGTIMSVR 302
  T ss_dssp_6VBG     HCCCCCCHHHHHTTSHHHHHHTTCSSSHHHHHHHHHHHHHHHHHHHHHHHHHH-----HHHHHSCHHHHHHHHHHHHHHH

  Q ss_pred_0325      HHHHHHccCHHHHHHHHHHHHHHHHHHHHHHHHHHHHhcccCCCCCHHHH-HHHHHHHHHHHHHHHHHHHHhCCccchhH
  Q MMSYN1_0325 333  VILITFIHNIYLLTIMMWVFGLFFFVMFYLWFGIALMWDYRSTKVSVLST-FLTVTFLTLSIWYLVISICKVNNIGLFSI 411
  T 6VBG_A      303  IIGSSFATSALEVVILKTLHMFEVPFLLVGCFKYITSQFEVRFSATIYLVCFCFFKQLAMIFMSVLAGNMYESIG----- 377
  T ss_dssp_6VBG     HHHHHHCCSHHHHHHHHHTHHHHHHHHHHHHHHHHHHHSCGGGHHHHCCCCCCCCHHHHHHHHHHHHHHHHHHSC-----

  Q ss_pred_0325      hccHHHHhhccchhhHHHHHHHHHHHHHHHHHHHHHHHHHHHHHHHHHHHHHHhcCCHHHHH
  Q MMSYN1_0325 412  FKSVFEVINNTDLNKNYLFIKKITEVYYICCILIFCLLGIYLTTFIWTANYIIAEYMDLKQIK 474 (496)
  T 6VBG_A      378  -----------------------FQGAYLVLGLVALGFTLISVFTLSGPGPLSLLRRQVNEVA 417 (417)
  T ss_dssp_6VBG     -----------------------HHHHHHHHHHHHHHHHHHHHHHSSSCCSGGGC--------
```

Supplemental Figure 1a.

```
  DSSP 0249  lllllLLLLLLLLEEEEEE-EEEELllllllllllllllhhhhhhlllllllllLLLLEEEEEEEELLEEEEELL---LLEEEEEHHHEEEEEEEEEE
  0249       eidfkYQLPENEIGLFQKE-LLISGinqkikdykdyfdddylvlkkslslyqttSYDFKQVKLYLTNLHLVIDDN---NQFYKYKIIEIKSCSICVIR
  2HTH       fvwtsGLLEINETLVIQQRgVRIYD--------------------------geeKIKFDAGTLLLSTHRLIWRDQknhECCMAILLSQIVFIEEQAAG
  DSSP 2HTH  leellLLLLLLLLEEEEEEeEEEEE--------------------------lllLLLLLLEEEEEELLEEEEEELlllLLLEEEEHHHEEEEEEEELLL

  DSSP 0249   elLEEEEEEEEEELL---------------LEEEEEL---LLHHHHHHHHHHLLL--l
  0249        dkNLLQKGCVLKTND---------------QSLTILG---DVFLLVLSIKKLKKE--f
  2HTH        --IGKSAKIVVHLHPappnkepgpfqssknSYIKLSFkehGQIEFYRRLSEEMTQrrw
  DSSP 2HTH   --LLLLEEEEEELLllllllllllllllllLEEEEEElllLHHHHHHHHHHHHHHlll
```

Supplemental Figure 1b.

```
DSSP 0332  LLLLHHHHHHHHHHHHHHL------LLL-LLEEEEE-LLLLLLhhhLEELL-------------LLEEEEELL----LLLLHHHHHHHHHHHHHHHHHHHLLL
0332       LVINSTDINKAIRQFQVMF------NLT-DYQIIYT-DTDNMIKvfkNINKN-------------KKQIISKR----IFESVGYELDYLISRLWISAKQVKKD
3E11       VYVDPDRFDELVAEALDGIpeefarAXR-NVAVFVEdEPDDPEL-lgLYVGIplterttayggvlPDRIIIYRNticaLCETESEVIDEVRKTVVHEIAHHFGI
4JIX       --ENKKDIKDIVNEILISL------NINeSINIEIKPMKQKI----aSFSFK-------------TKTLRLNKY------vvenFDEELLHYIILHELIHFKIK
6CZ6       ESGXRTGGLDIQLAELXRd------rFGISVVIDDn--LPDTA--krRYHPD-------------TKVLRVAHW------lXPGQRAFQIATQLALVGQSdliS
DSSP  3E11 lLLLHHHHHHHHHHHHHLLlhhhlhHHL-LEEEEEElLLLLLL-leEEELLlhhhlllllllllLEEEEEEHHhhhhLLLLHHHHHHHHHHHHHHHHHHHLLL
DSSP  4JIX --LLLLLHHHHHHHHHHHL------LLLlLLEEEEELLLLLL----eEEELLLL-----------EEEEEHHhh------hhLLHHHHHHHHHHHHHHHHHHLLL
DSSP  6CZ6 hllllllLHHHHHHHHHHh------hHLLEEEEELl--LLLLE--eeEEELL-------------LLEEEEELL------LLHHHHHHHHHHHHHHHHLHLLLL
```

Supplemental Figure 1c.

```
DSSP 0338 LLHHhhLLLLEEeELLLLLHHhhHHHHHhhLLLLLhhHHLLEEEEEEEEELllllllllllllLLEEEEEEEEELLllllLLEEEEEEEEEL
0338      IDLNklIKVRDLgFVSKNEKEiiKSAFVkqNGLNDpkLKDKIEVEVKTNGSgtsgagttastngnSSDSAVIEVKNKTngngNVTKTVTVIFDV
6SX1      DKYT--PEAKDITVTPgPTPDPAEGIGN------KDTLPSGTKYEWKDPVD----------tttpGDKTGTIVVSYPD----GSTDEIQVTVKV
DSSP 6SX1 HLLL--LLLLLEEELLlLLLLHHHHLLL------HHHLLLLLEEEELLLLL----------llllEEEEEEEEEELLL----LLEEEEEEEEEE

DSSP 0338 hhHHLLLLEEEEELLLLhhHHHHHHHHHL----HHHL---lLHHHEEEEEEELL---------------EEEEEE------llllLLLL--LEEEEEEELL
0338      lkTLVKVTKLKSLPDNKdeTILAAVAKAN----PKSN---lDTQKLKIERTDG---------------KVLVKS------sdgqTYKD--EAELQIESKV
6QX4      laVEFTSTSLKEVAPNA--DLKAALLNILsvdgVPATtakaTVSNVEFVSADTnvvaengtvgakgatSIYVKNltvvkdgkeqKVEFdkAVQVAVSIKE
DSSP 6QX4 llEEELLLEEEEELLLL--LHHHHHHLLEeellEELLllllLEEEEEEEELLLllllllllllllleeEEEEEEeeeeelleeeEEEEeeEEEEEEEELL
```

Supplemental Figure 1d.

```
DSSP 0392 LLLHHHHHHHHHHHHHhhlllllhhheEEEEEEEL----------------------LLEEEEEEEEELL-lllllLLLLHHHHHHHHHHHHHHHHLLLEEEEEEEEEL
0392      KIESKVINRLVENVILsmtkisdpknvSSSIYVLD----------------------ENQLHILATIKIG-deklqDLNINEDKIFKAIDKTINQTISMKPKNINISYIR
5D6J      NHYPQDLEYSAQEASK-----airtgYVAAFSVPanqlpdevfenahsgikrdpddtSEQLVIVAERAPG------AHKLDIGPITDDIRAAIAVRHGVTVRDVLLTAAG
6HAN      GSHMEAVQNRIVEAAER-VPGVrgvIHLRARYVG-----------------------QDIWADMIIGVd------PENTVEQaeEICEAVQAAVCGKIRRIESLHVSAHA
DSSP 5D6J EELHHHHHHHHHHHLL-----lllllLEEEEEEEhhhllhhhhhlhhhlllllllllLEEEEEEEELLLL------LLLLLHHHHHHHHHHHHHHHHLLLEEEEEEELLL
DSSP 6HAN LHHHHHHHHHHHHHHHL-ьLLLLlleEEEEEEEEL----------------------LEEEEEEEEEEl------LLLLHHHhhHHHHHHHHHHHHHLLLEEEEEEEEEE
```

Supplemental Figure 1e.
