# Supplementary material for: Minimal organism JCVI-syn3.0, an engineered derivative of Mycoplasma mycoides, encodes an assortment of unrecognized membrane transporters and immunity factors
Source: mSphere. 2026 Aug 28;11(9):e00077-26. doi: 10.1128/msphere.00077-26 (PMC13621843; doi:10.1128/msphere.00077-26)
Supplement: Supplementary file 2 [file msphere.00077-26-s0002.pdf]

8BAR E. coli C7 DarT1; TOXIN-ANTITOXIN, DNA ADP-RIBOSYLATION, ADP-ribosyltransferase activity, DNA binding, TOXIN; HET: EDO, APR; 1.63A {Escherichia coli} Probability: 98.06%, E-value: 0.00012, Score: 61.41, Aligned cols: 137, Identities: 16%, Similarity: 0.294, Template Neff: 9.2

```
Q ss_pred_0281       hhHHHHHHhCCCCEEEEEecHHHHHHHHHhCceeccccCCC-----CceEEEEEEeccCCeeeeeccccchhHHHHHHHh
Q MMSYN_0281    54  KNVLDFINKQDIQYFLFYTNSKNWLNILQYGICPVKEIKLKA-----DEEYVVWSFQQKDYSIGLAFDISSRAQFWKWLK 128
T 8BAR_A         2  MTIQEIIQQRNIRSLFHFTHSDNLTSILDNGLMSRSELDNENNEYNCNDEE---RIDGHPDAICLSVSYPNAKMFYKYRC  78
T ss_dssp_8BAR      CCHHHHHHHHTCCCEEEEEEGGGHHHHHHHCBCCHHHHHHTTCCCCCCCSS---CTTSCTTCEEEEESSCCHHHHHHHHH

Q ss_pred_0281       cCCCChhheEEEEECHHHHHHh-----------------------hhhhhh--hc------------------cccCeeE
Q MMSYN_0281   129 DTDIKTDQFLTIAINPNTLYRV-----------------------TKKDWV--WD------------------KSLSMVF 165
T 8BAR_A        79 LKP---GDWVILEINPSVLWAKDCAFYPTNAASNNVRFINLDLMKGAEAFSALFSENVFGIQRDVNLPSEYTTDVQAEIL 155
T ss_dssp_8BAR      HSC---SCEEEEEECTTHHHHSCEEEESSCTTSTTTTTSCGGGGBHHHHHHGGGCSEETTEECCTTSCTTSCSSTTCEEE

Q ss_pred_0281       EeccccCHHhEEEeEecCHHHHHHHHHHHHHc
Q MMSYN_0281   166 INEAIQIECIEWILFRDYDLYKKAEEYLRKT 196 (226)
T 8BAR_A       156 VFEKIPPSYIISTFHPNKESAEHFKRLYPQT 186 (208)
T ss_dssp_8BAR      ESSCBCGGGEEEEEESSHHHHHHHHHHCTTS
```

Supplemental Figure 2a. HHPred alignment of the sequence models for MMSYN1_0281 (EBI A0A7Z7LCS0) and toxin DarT1 from *E.coli*. In the secondary structure lines (ss), H indicates a helix and E indicates elongated structure (a strand). The four residues directly involved in catalysis in DarT toxins (Schuller et al., 2023) are indicated by cyan shading.

Fic-domain containing protein; Toxin, Fic protein, Cytidylyltransferase, nucleotidyltransferase activity; HET: EDO, CDP, ACT, SO4; 2.6A {Mycobacterium marinum (strain ATCC BAA-535 / M)}Probability: 100%, E-value: 5.3e-32, Score: 264.05, Aligned cols: 297, Identities: 12%, Similarity: 0.069, Template Neff: 10.1

```
Q ss_pred_0805   HHHHHHHHHHHHHHHHHHhccCcCCcchhhHHHHHHHHHcCCCCCcHHHHHHHHHHHHHHHHHhcC-C--CCCCHHHHHHHH
Q MMSYN_0805  63 ESSVEKKLKTYELALLVMNETKIDLDFLSVLKIFRDIKKGKTPTNYLERLIFNIIYAYEYIKKP-K--VLINEENLEMLI 139
T 7CB8_A      75 LAGLSTLLLRTESVASSKIERVE----AS-VDDYARALHGGRG-NSSAVSMVAATTALKEMIASVNRDAPIQMTAILRAH 148
T ss_dssp_7CB8   CTTHHHHHHHHHHHHHHHHHTTCC----CC-HHHHHHHHTTCCS-CHHHHHHHHHHHHHHHHHHHTSSSSCCCCHHHHHHHH

Q ss_pred_0805   HHHHcCCCCCcccccCCccccCCCCccc---------cccCCCHHHHHHHHHHHHHHHHHHhhhcCCCHHHHHHHHHHHHHHH
Q MMSYN_0805 140 SILLVGLEYDLDLKTNYYRTPKTKTL---------ISNVLSSQLISKELENLLDYLKFLQANNLCTYSQTYLIFSTLVLI 210 (391)
T 7CB8_A     149 EALMREDPTE-GQHAGQVRTVQNWIGGSDYSPRNALYVPPPPDTVHAYMDDLIEFANR---TDIPVLIQAAIAHAQFESI 224 (412)
T ss_dssp_7CB8   HHHHTTCTTT-GGGTTSCCCSCCCBSSCSSSSTTCSBCCCCGGGHHHHHHHHHHHTTC---SSSCHHHHHHHHHHHHHHHH

Q ss_pred_0805    CcCcccChHHHHHHHHHHHHHHHcCCCccccceeehH-HHhcchHHHHHHHHHHHHcCCCCHHHHHHHHHHHHHHHHHHHchHHH
Q MMSYN_0805 211  SPFQKYNLIFATLLSQWISFQYNNSYKLVIPIC-HFLKNQNEYMYELENLLNNDFNADKLINLFNIDYLKNINMYNHASC 289 (391)
T 7CB8_A     225  HPFTDGNGRIGRALINTVLRRRGATTRLVVPLASALVAHRERYFGALNTYRA--GDLRPLIVTFANSSRTAAAESRIT-- 300 (412)
T ss_dssp_7CB8    CCSSSCHHHHHHHHHHHHHHHHTTSCSSCCCCTHHHHHHTHHHHHHHHHHHHHT—TBCHHHHHHHHHHHHHHHHHHHHHHH--

Q ss_pred_0805   HHHH--HHh----cCccccccchhhHHHHHHHHHHcCCCceeHHHHHHHhhhccccCCCHHHHHHHHHHHHHCCCeeecC-
Q MMSYN_0805 290 IYKW--VKK----DKKRLFIFEDDLSFFVLILILQNTKNLSFNNIKTLLTINKIKLFTDEQIKSTLANLIANQVLQTTS- 362 (391)
T 7CB8_A     301 AERLAEIPVEWRNMVGPIRRHSATD---KLLLLLPSTPIVSSDDVASLI------DAPRSSVFAAIKRLHDTGVLRPLTN 371 (412)
T ss_dssp_7CB8   HHHHHHHHHHHHHHHHCCCCTTSHHH---HHHHHTTTCCEEEHHHHHHHS------SSCHHHHHHHHHHHHHHHTSEEESCC

Q ss_pred         C-CeEEEEechHHHHHHHHHHH
Q MMSYN_0805 363 T-SVVKYVLVDKYLEKSKYLVN 383 (391)
T 7CB8_A     372 RKRDQVWGASL-VLDELDDLGH 392 (412)
T ss_dssp_7CB8   C-CCCEEEEHH-HHHHHHHHHH
```

Supplemental Figure 2b.
