# Supplementary material for: Minimal organism JCVI-syn3.0, an engineered derivative of Mycoplasma mycoides, encodes an assortment of unrecognized membrane transporters and immunity factors
Source: mSphere. 2026 Aug 28;11(9):e00077-26. doi: 10.1128/msphere.00077-26 (PMC13621843; doi:10.1128/msphere.00077-26)
Supplement: Supplementary file 3 [file msphere.00077-26-s0003.pdf]

Eco29kIR; type II restriction endonuclease, GIY-YIG endonuclease, HYDROLASE; 2.3A {Escherichia coli}
Probability: 65.37%, E-value: 130, Score: 25.79, Aligned cols: 94, Identities: 19%, Similarity: 0.115, Template Neff: 6.6

```
Q ss_pred 0777   hHHHHHHHHccCccchhHhhcccccccEEEEEEecCCCCCC--------CCccEEEEEecC-----------------
Q MMSYN1_0777 12 YKKVLNLLSTRQAYSWTQIDNIDQQGYFLIGITLKDSNYNK--------EKPLITLLKITD-----------------  64 (123)
T 3MX1_A      43 IKDAVRFFSGTPVHSLPPPERFQGAGVYALYYTGHYSLYDEYSRINRLAYNLPIYVGKAVPAGWRQSRISDHETRAGS 120 (235)
T ss_dssp 3MX1   HHHHHHHHHTSCCEESSCSSCCCCCEEEEEEECSCCGGGTTHHHHTSSSCCCCSEEEEECCTTTSCSSCSSCSSCCCS

Q ss_pred 0777   -HHHHHHHHHHHHhHHHHHHHHHHHCCCChhhEEEEEEEeCC----ChHHHHHHHHHHHhhhhc-
Q MMSYN1_0777 65 -LKTDISRFKSNINDYKNIINYLKQYNLTTKDLVFIIIEKVE----NSDELDKLLIEWNSLISA- 123 (123)
T 3MX1_A     121 ELSNRIREHGRNIAKTS---------NLDLCDFSCRFVIFEATGSDMISTVEAALIKKIYKPLWN 175 (235)
T ss_dssp 3MX1   HHHHHHHHHHHHHHTCS---------SCCGGGEEEEEEECCSGGGGGHHHHHHHHHHHHHHHHHH
                   *   *                                          *      ?     *
```

Additional Figure 3.
