# Supplementary material for: Minimal organism JCVI-syn3.0, an engineered derivative of Mycoplasma mycoides, encodes an assortment of unrecognized membrane transporters and immunity factors
Source: mSphere. 2026 Aug 28;11(9):e00077-26. doi: 10.1128/msphere.00077-26 (PMC13621843; doi:10.1128/msphere.00077-26)
Supplement: Supplementary file 4 [file msphere.00077-26-s0004.pdf]

Supplemental Files Legend

Supplemental Figure 1.

A. HHPred alignment of the sequence models for MMSYN1_0325 (EBI A0A7Z7LBY4) and galactoside permease LacY from *E.coli*. In the secondary structure lines (ss), H indicates a helix.

B. DALI alignment of MMSYN1_0249 (EBI A0A7Z7LCQ6) and PH-domain of human VPS36 (PDB 2HTH). Helical regions (H) and elongated/strand regions (E) are indicated by red and blue type, respectively. Conserved hydrophobic residues are yellow shaded.

C. DALI alignment of MMSYN1_0332 (EBI A0A7U0XNG8) and zinc peptidase-like domains in bacterial metalloprotease (PDB 3E11), archaeal jannalysin (PDB 4JIX) and *Mycobacterium tuberculosis* transcriptional regulator PrpR (PDB 6CZ6). Helical regions (H) and elongated/strand regions (E) are indicated by red and blue type, respectively. Conserved hydrophobic residues are yellow shaded, and the catalytic active center residues in 3E11 and 4JIX are cyan shaded.

D. DALI alignments of MMSYN_0338 N-terminal and C-terminal regions (EBI A0A7Z7LBE2) with two distinct immunoglobulin-like domains in Gram-positive bacteria. Top, alignment of MMSYN_0338 N-terminal region and Rib domain of *Lactobacillus acidophillus*. Bottom, alignment of MMSYN_0338 C-terminal region and *Bacillus antracis* S-layer protein Sap. Red and blue type indicates, respectively, helical regions (H) and elongated/strand regions (E). Conserved hydrophobic residues are yellow shaded, other conserved residues are gray shaded.

E. DALI alignment of MMSYN_0392 (EBI A0A014MFN5) and two small molecule-binding domains with the alpha-lytic protease prodomain-like fold. Helical regions (H) and elongated/strand regions (E) are indicated by red and blue type, respectively. Conserved hydrophobic residues are yellow shaded.

Supplemental Figure 2.

A. HHPred alignment of the sequence models for MMSYN1_0281 (EBI A0A7Z7LCS0) and toxin DarT1 from *E.coli*. In the secondary structure lines (ss), H indicates a helix and E indicates elongated structure (a strand). The four residues directly involved in catalysis in DarT toxins are indicated by cyan shading.

B. HHPred alignment of the sequence models for MMSYN1_0805 (EBI A0A7U1AI95) and a stand-alone Doc/Fic family protein CccR from *Yersinia pseudotuberculosis*. In the secondary structure lines (ss), H indicates a helix and E indicates elongated structure (a strand). The conserved Doc/Fic motif in CccR, with the histidine residue implied in catalysis in several members of the family, is indicated by cyan shading.

Supplemental Figure 3. HHPred alignment of the sequence models for MMSYN1_0777 (EBI AF-A0AB38GEN3-F1-v6) and GIY-YIG endonuclease R.Eco29kI from *E.coli* (3MX1). In the secondary structure lines (ss), H indicates a helix and E indicates elongated structure (a strand). The signature related to the GIY-YIG pattern and partially replaced in 3MX1 is shaded in yellow. Four residues directly involved in interactions with DNA and catalysis in 3MX1 and also partially replaced in MMSYN1_0777 are indicated by cyan shading and asterisks.

Supplemental Table 1. Identifiers and sequences of proteins in Set C.

Supplemental Table 2. Protein functions predicted in this study compared to several predictions published in 2018-2025
